# Integration of Bioinformatics, Serum Pharmacochemistry, and Metabolomics to Reveal the Mechanisms of Danggui Buxue Decoction in Anti‐Rheumatoid Arthritis Through Inflammation and NF‐κB Signaling Pathway Regulation

**DOI:** 10.1002/iid3.70259

**Published:** 2025-09-16

**Authors:** Lianyun Du, Ye Zhai, Meixiu Luo, Lu Tang, Saiyue Qiu, Xin Jiang, Enpeng Wang, Zhi Pan

**Affiliations:** ^1^ Jilin Ginseng Academy Changchun University of Chinese Medicine Changchun China; ^2^ College of Traditional Chinese Medicine Changchun University of Chinese Medicine Changchun China; ^3^ College of Integrative Medicine Changchun University of Chinese Medicine Changchun China

**Keywords:** bioinformatics analysis, Danggui buxue decoction, inflammation, metabolomics, NF‐κB signaling pathway, rheumatoid arthritis, serum pharmacochemistry

## Abstract

**Objective:**

Rheumatoid arthritis (RA) remains a significant clinical challenge due to the limited efficacy and adverse effects associated with conventional treatments. Danggui buxue decoction (DBD), renowned for its qi‐tonifying, blood‐nourishing, and anti‐inflammatory properties, has shown promising potential in the management of RA. This study investigates the mechanisms of DBD in RA through bioinformatics and experimental analyses, providing a scientific foundation for its clinical application and further development.

**Methods:**

The chemical components and blood‐absorbed components of DBD were identified using UPLC‐MS/MS. Bioinformatics approaches were applied to identify differentially expressed genes (DEGs) between RA patients and healthy individuals, and to predict potential drug targets and signaling pathways associated with the blood‐absorbed components of DBD. Collagen‐induced arthritis (CIA) rat models and tumor necrosis factor (TNF)‐α‐stimulated human rheumatoid arthritis fibroblast‐like synoviocyte (RA‐FLS) models were established to evaluate the therapeutic effects of DBD on RA. Serum metabolomics analysis was performed to identify differential metabolites and key metabolic pathways associated with the therapeutic effects of DBD.

**Results:**

A total of 83 chemical components and 34 blood‐absorbed components were identified in DBD. Bioinformatics analysis highlighted the NF‐κB signaling pathway as a central regulator of RA‐related inflammation, requiring further mechanism exploration. Experimental findings demonstrated that DBD significantly reduced the expression of inflammatory cytokines‐IL‐1β, IL‐6, and TNF‐α‐in both RA‐FLS cells and serum from CIA rats. Furthermore, DBD inhibited RA‐FLS cell proliferation and migration while inducing apoptosis. DBD treatment alleviated paw swelling, reduced arthritis scores, and decreased spleen and thymus indices. Histopathological examination (HE) staining showed decreased inflammatory cell infiltration, and Micro‐CT results revealed notable mitigation of bone destruction. Metabolomic analysis identified 34 differential metabolites and 4 significant metabolic pathways (*p* < 0.05). Moreover, DBD significantly downregulated the expression of phosphorylated proteins in both RA‐FLS cells and synovial tissue of rats involved in the NF‐κB signaling pathway, including p‐IκBα, p‐p65, and p‐IKKα, and suppressing NLRP3 protein and mRNA expression of IκBα, p65, and IKKα.

**Conclusion:**

DBD effectively alleviates RA symptoms by reducing inflammation and inhibiting the activation of the NF‐κB signaling pathway through its multi‐component and multi‐target characteristics. This study provides valuable insights into the molecular mechanisms underlying the anti‐RA effects of DBD.

## Graphical abstract

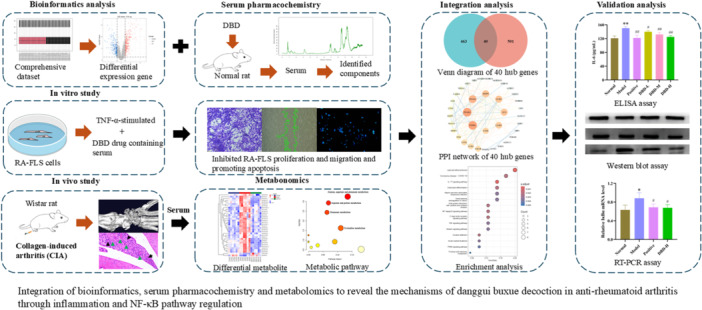

## 1 Introduction

Rheumatoid arthritis (RA) is a chronic autoimmune disease characterized by progressive inflammation of the synovial membrane and subsequent bone destruction, with a global prevalence ranging from 0.5% to 1.0% [1, 2]. Current therapeutic strategies have expanded from traditional nonsteroidal anti‐inflammatory drugs (NSAIDs), glucocorticoids, and disease‐modifying anti‐rheumatic drugs (DMARDs) to include targeted biological agents aimed at specific cytokines, such as tumor necrosis factor (TNF)‐α, cyclooxygenase (COX), and interleukins (IL) [3, 4, 5, 6]. Despite these advances, the clinical application of existing therapies remains limited by adverse immune responses and significant side effects [7]. Thus, there is an urgent need for novel complementary or alternative therapeutic drugs for RA.

In traditional Chinese medicine (TCM), RA is classified as a “Bi syndrome,” caused by a deficiency of vital Qi and the invasion of pathogenic factors like wind, cold, dampness, and heat, which obstruct the flow of Qi and blood and lead to blood stasis formation in the meridians [8, 9, 10]. TCM has developed a wealth of clinical experience and effective prescriptions for anti‐RA. One such formula is danggui buxue decoction (DBD), a classical herbal prescription composed of A*stragalus membranaceus* (Fisch.) Bunge (Fabaceae) radix (Huangqi) and *Angelica sinensis* (Oliv.) Diels (Apiaceae) radix (Danggui) at a ratio of 5:1. It has been used in China for over 800 years [11]. Li Shizhen, a renowned ancient Chinese physician, referred to Huangqi as the “prince of qi‐supplementing herbs”, emphasizing its function in greatly replenishing the qi of the spleen and lungs, thereby promoting blood production [12]. Meanwhile, Danggui is primarily known for its effects of nourishing blood, regulating blood circulation, and promoting meridian flow. When used together, these two herbs exert a synergistic effect on both qi and blood, enhancing blood circulation and exerting a blood‐activating and stasis‐resolving effect [13]. DBD has demonstrated anti‐inflammatory effects and is utilized in the treatment of inflammation, immune‐related disorders, and imbalances in Qi and blood [14, 15]. It is also commonly used in the clinical management of RA, however, experimental studies on DBD in anti‐RA therapy remain relatively limited, the specific chemical components, blood‐absorbed components, and the modern pharmacological mechanism underlying DBD's therapeutic effects on RA have not been fully studied.

**Figure 1:**
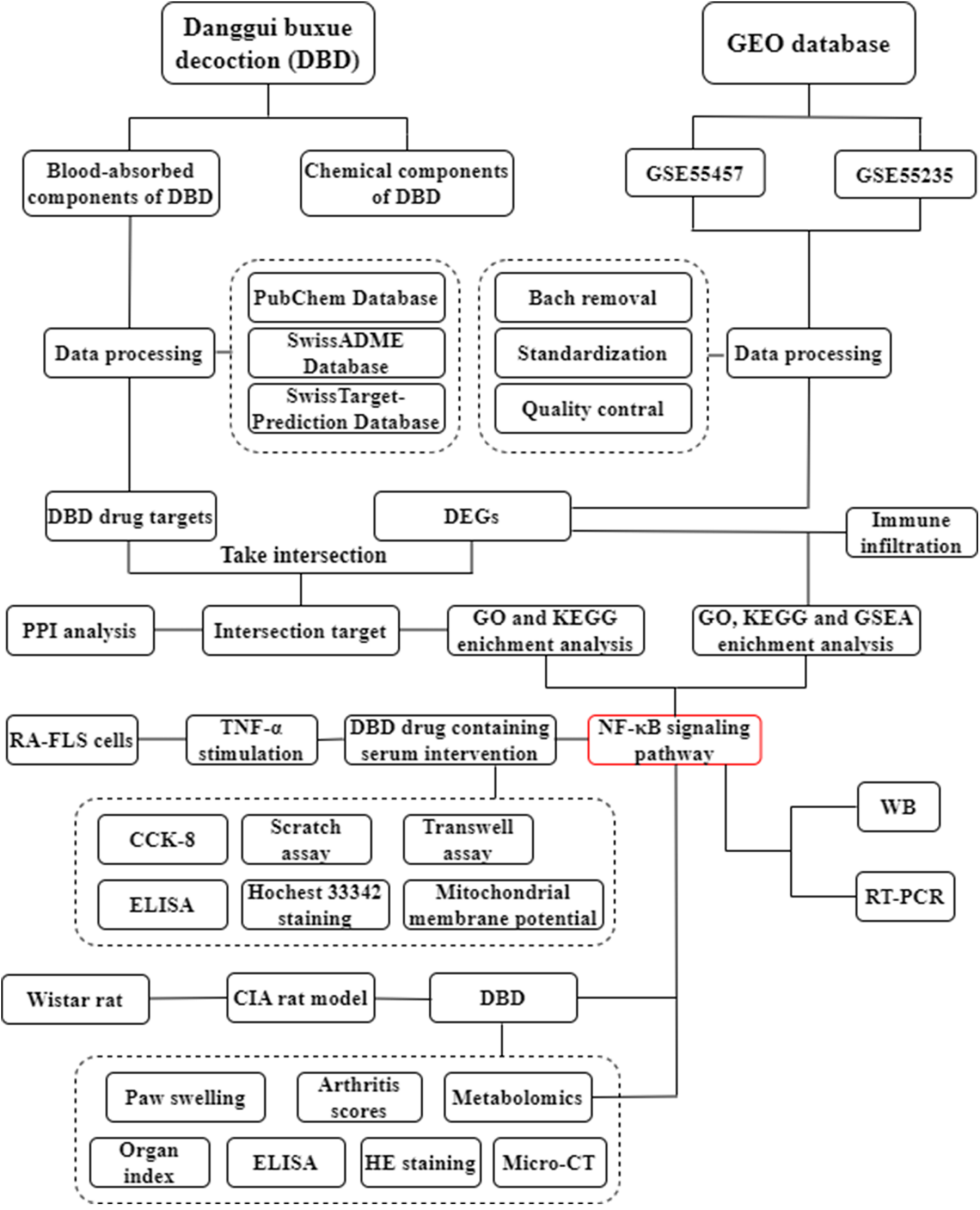
Technical strategy of the current study.

In recent years, bioinformatics, serum pharmacochemistry and metabolomics have become essential tools for exploring the mechanisms of TCM. Bioinformatics aids in the identification of disease‐related targets and key signaling pathways through systematic analysis of molecular networks [16], while serum pharmacochemistry provides in‐depth insights into the active components of herbal formulations and their potential biological activities [17]. Metabolomics, as a high‐throughput analytical technique, has been widely applied in areas such as biomarker discovery, disease diagnosis, mechanism exploration, prognosis prediction, treatment monitoring, and drug development [18, 19]. However, the integrated application of these three techniques has not yet been fully explored in the context of DBD in anti‐RA, presenting an opportunity for further investigation. In this study, bioinformatics, serum pharmacochemistry, and metabolomics were combined to identify the therapeutic targets and pathways of DBD in anti‐RA. Our findings were validated through both in vivo and in vitro experiments, providing a theoretical basis for the clinical application of DBD. The technical strategy of this study is shown in Figure 1.

## 2 Materials and Methods

### 2.1 Chemicals and Reagents

Huangqi and Danggui were purchased from Hongjian Pharmacy (Changchun, China), which also provided authentication of the herbal materials. The positive drug tripterygium glycosides tablets were purchased from Shanghai Fudan Fuhua Pharmaceutical Co. Ltd. The bovine type II collagen (CII) was provided by Chondrex Inc. (Chondrex, Seattle, WA, USA). Complete Freund's adjuvant (CFA) was provided by Sigma‐Aldrich Co. (St. Louis, MO, USA). Glacial acetic acid was obtained from Beijing Chemical Plant. The paraformaldehyde was obtained from Shanghai yuanye Bio‐Technology Co. Ltd. (Shanghai, China).

Fibroblast medium (FM), fibroblast growth supplement (FGS), fetal bovine serum (FBS) and penicillin/streptomycin solution (P/S) were purchased from ScienCell Research Laboratories (California, USA). Human rheumatoid arthritis fibroblast‐like synoviocytes (RA‐FLS) cells were obtained from the Shanghai Guandao Biological Engineering Co. Ltd (Shanghai, China). The ELISA assay kits for rat and human TNF‐α, IL‐1β and IL‐6 were all obtained from Shanghai Sino Best Biological Technology Co. Ltd. (Shanghai, China). Cell counting kit‐8 (CCK‐8) was purchased from Biyuntian Biotechnology Co. Ltd. (Shanghai, China). Total RNA extraction kit was purchased from Beijing Tiangen Biochemical Technology Co. Ltd (Beijing, China). The antibodies of IκBα, p‐IκBα, p65, p‐p65, IKKα, p‐IKKα (Batch number: A16929, AP0707, A19653, AP0944, A2062 and AP0505, respectively) were purchased from Wuhan ABclonal Biotechnology Co. Ltd. and NLRP3 (Batch number: 30109‐1‐AP) were purchased from Wuhan Sanying Biotechnology Co. Ltd. (Wuhan, China).

### 2.2 Preparation of DBD

Based on the *Chinese Pharmacopoeia* (2020 edition) and the *Pharmacological Experimental Methodology*, the human‐to‐animal equivalent dose and body surface area scaling determined the rat equivalent dose of DBD as 7.5 g/kg (Middle dose), with low and high doses set at 3.75 g/kg and 15.0 g/kg, respectively. Huangqi and Danggui (5:1, w/w) were soaked in distilled water (1:10, w/v) at room temperature for 30 min, then decocted for 45 min. The filtrate was collected, and the residue underwent a second decoction in distilled water (1:8, w/v) for another 45 min. The combined filtrates were concentrated to prepare solutions for low (0.375 g/mL), middle (0.75 g/mL), and high (1.5 g/mL) dose groups. The positive group, triptolide glycosides, was prepared at 0.945 mg/mL. All solutions were administered via intragastric administration at 10 mL/kg.

### 2.3 Analysis of Chemical and Blood‐Absorbed Components of DBD

#### 2.3.1 Preparation of Drug Containing Serum

12 male wistar rats (180 ± 20 g) were provided by the Changchun Yisi Laboratory Animal Technology Co. Ltd. (Certificate No. SCXK [Ji] 2018–0007, Changchun, China). All animal experimental protocol adheres to the guidelines for international animal experiments approved by the Animal Management and Use Committee of Changchun University of Chinese Medicine (Animal ethics approval number: 2025365). Rats were kept under normal lighting conditions with room temperature maintained at 22°C–25°C.

The preparation of drug‐containing serum for detecting blood‐absorbed components was carried out as follows: Following an adaptive feeding period, randomization was performed using a random number generator to assign animals to different groups, ensuring unbiased distribution. Efforts were made to conduct the experiments in a single‐blind manner where feasible to minimize observer bias, 12 rats were randomly divided into a normal group and a high‐dose DBD (DBD‐H, 15.0 g/kg) group (*n* = 6 per group). Rats in the DBD‐H group received the decoction and rats in normal group were given distilled water via intragastric administration once daily at a fixed time for seven consecutive days. Blood samples were obtained from the abdominal aorta after anesthesia with 20% ethyl carbamate, and serum was separated by centrifugation. The samples from the same group were then pooled and homogenized before being stored at −80°C for subsequent analysis.

#### 2.3.2 Preparation of Samples and UPLC‐MS/MS Conditions

DBD was prepared as freeze‐dried powder for subsequent component analysis. The appropriate amount of freeze‐dried powder and drug‐containing serum of DBD was mixed with 400 μL of methanol and vortexed, respectively. The mixture was then centrifuged, and the supernatant was collected, concentrated, and dried. The residue was reconstituted using 2‐chloro‐l‐phenylalanine (4 ppm) and filtered through a 0.22 μm membrane for UPLC‐MS/MS analysis.

Liquid chromatography was performed on a Vanquish UHPLC system with a Waters ACQUITY UPLC HSS T3 column at 40°C, coupled to a Thermo Fisher Q Exactive mass spectrometer in positive and negative ion modes. In the positive ion mode, the mobile phases were 0.1% formic acid acetonitrile (B1) and 0.1% formic acid water (A1), with the gradient elution program as follows: 0–1 min, 8% B1; 1–8 min, 8%–98% B1; 8–10 min, 98% B1; 10–10.1 min, 98%‐8% B1; 10.1–12 min, 8% B1. In the negative ion mode, the mobile phases were acetonitrile (B2) and 5 mM ammonium formate water (A2), with the gradient elution program as follows: 0–1 min, 8% B2; 1–8 min, 8%–98% B2; 8–10 min, 98% B2; 10–10.1 min, 98%–8% B2; 10.1–12 min, 8% B2. Mass spectrometry conditions: Scan range: m/z 100–1000, positive ion spray voltage: 3.50 kV, negative ion spray voltage: −2.50 kV, sheath gas flow rate: 40 arb, auxiliary gas flow rate: 10 arb, capillary temperature: 325°C.

### 2.4 Bioinformatics Exploration of the Anti‐RA Mechanism of DBD

#### 2.4.1 Acquisition and Processing of RA Disease Data Set

Two gene expression datasets, GSE55457 and GSE55235, were selected from the GEO database (https://www.ncbi.nlm.nih.gov/geo/). The GSE55457 data set (Platforms: GPL96[HG‐U133A] Affymetrix Human Genome U133A Array; BioProject: PRJNA240150; Public: Mar 05, 2014 and Last update date: Aug 10, 2018) includes sequencing data from synovial tissues of 10 healthy individuals and 13 RA patients, while the GSE55235 data set (Platforms: GPL96[HG‐U133A] Affymetrix Human Genome U133A Array; BioProject: PRJNA238979; Public: Feb 21, 2014 and Last update date: Aug 10, 2018) comprises 10 healthy individuals and 10 RA patients. The “SVA” package in R software was used to standardize data quality and eliminate batch effects from the matrix data, enabling the identification of therapeutic targets for RA.

#### 2.4.2 Data Processing and Differential Expression Genes (DEGs) Screening

The “Limma” package in R software was used to identify DEGs from the GSE55457 and GSE55235 datasets, with the screening criteria set as follow: |logFC | ≥ 1, *p* < 0.05. The “pheatmap” and “ggplot2” packages in R were employed to visualize the DEGs. Finally, the data from the two datasets were integrated, and DEGs between healthy individuals and RA patients in synovial tissues were subjected to principal component analysis (PCA) analysis. The volcano plot and heatmap of the DEGs were generated.

#### 2.4.3 Enrichment Analysis of DEGs

Based on previously DEGs, the “GenetrProfile” package in R software was used for Gene Ontology (GO) analysis, Kyoto Encyclopedia of Genes and Genomes (KEGG) analysis, and Gene Set Enrichment Analysis (GSEA). The top 15 enrichment terms were selected and visualized as output.

#### 2.4.4 Analysis of Immune Infiltrating Cells of DEGs

Based on the DEGs data matrix analysis results, the “X cell” package in R software was used to analyze the immune infiltration of DEGs, and the expression differences of 67 immune cells between different groups were compared. The “ggplot2” package in R software was used to draw the box plot visualization analysis results.

#### 2.4.5 Acquisition of Drug Targets of Blood‐Absorbed Components in DBD

To analyze the target of DBD, PubChem (https://pubchem.ncbi.nlm.nih.gov/), Swiss ADME (http://www.swissadme.ch/index.php), and Swiss Target Prediction (http://www.swisstargetprediction.ch/) were utilized. Structural data in SDF or SMILES format were obtained from PubChem for standardized modeling. Swiss ADME was used to screen components with favorable pharmacokinetics (Components with high GI absorption, compliance with Lipinski, Veber, and Egan rules, and a bioavailability score ≥ 0.55) [20]. Targets with a prediction probability > 0 were identified via Swiss Target Prediction for further analysis.

#### 2.4.6 Enrichment Analysis of Hub Genes

The DEGs of RA identified in Section 2.4‐2 were cross‐referenced with the DBD drug targets identified in Section 2.4‐5 to obtain overlapping genes, which were recognized as hub genes for DBD in anti‐RA. GO and KEGG enrichment analysis were performed on the hub genes. The top 15 enrichment terms were selected and visualized as output.

#### 2.4.7 Construction of Protein‐Protein Interaction (PPI) Network

A PPI network for the hub genes was constructed using the “Multiple Proteins” function in the STRING database (https://cn.string-db.org/), with “Homo sapiens” specified as the target organism.

### 2.5 In Vitro Study on the Anti‐RA Mechanism of DBD

#### 2.5.1 Preparation of Drug Containing Serum, Cell Grouping, and Intervention

The preparation of drug containing serum of DBD for cell intervention is as follows: Randomization was performed using a random number generator to assign animals to different groups, ensuring unbiased distribution. Efforts were made to conduct the experiments in a single‐blind manner where feasible to minimize observer bias. 36 wistar rats were randomly divided into 5 groups. 12 rats in the normal group were administered an equivalent volume of distilled water, and their serum was used for intervening cells in the normal and model groups (*n* = 12 in normal group). The remaining 24 rats were randomly divided into 4 groups and administered either a positive drug or different doses of DBD (DBD low‐dose: 3.75 g/kg, DBD middle‐dose: 7.5 g/kg, DBD high‐dose: 15.0 g/kg) (*n* = 6 in positive and different doses of DBD group). After 7 days of continuous intragastric administration, serum was collected using the above method, then inactivated at 56°C, and stored at −20°C for future use.

RA‐FLS cells were cultured at 37°C in FM medium supplemented with 10% FBS, FGS, and P/S solution under 5% CO₂ and 95% air. Then RA‐FLS cells were randomly divided into the following 6 groups: Normal group (Basal culture medium+10% normal group rat serum medium), model group (20 ng/mL TNF‐α + 10% normal group rat serum medium), positive group (20 ng/mL TNF‐α + 10% positive group serum medium), DBD low‐dose (20 ng/mL TNF‐α + 10% DBD low‐dose group rat serum medium, DBD‐L), DBD middle‐dose (20 ng/mL TNF‐α + 10% DBD middle‐dose group rat serum medium, DBD‐M), and DBD high‐dose (20 ng/mL TNF‐α + 10% DBD high‐dose group rat serum medium, DBD‐H).

#### 2.5.2 Cell Proliferation Assay

RA‐FLS cell proliferation was assessed using the CCK‐8 assay. 100 μL cell suspension (5 × 10³ cells/well) were seeded in 96‐well plates, incubated at 37°C for 24 h, and stimulated with TNF‐α (20 ng/mL) for 1 h. Cells were then treated with drug‐containing serum from different groups for 24 or 48 h. After adding 10 µL of CCK‐8 solution and incubating for 3 h, absorbance at 450 nm was measured to calculate the cell proliferation inhibition rate.

#### 2.5.3 Inflammatory Factors

Inflammatory factor levels in RA‐FLS cells were measured via ELISA. RA‐FLS cells (2.5 × 10⁵ cells/well) were seeded in 6‐well plates and incubated at 37°C for 12 h. After stimulation with TNF‐α (20 ng/mL) for 1 h and treatment with drug containing serum for 24 h, the supernatant was collected, and TNF‐α, IL‐6, and IL‐1β levels were determined following the ELISA kit protocol.

#### 2.5.4 Transwell Assay and Scratch Assay of Cell Migration

The Transwell assay was used to assess the effect of DBD on RA‐FLS cell vertical migration. RA‐FLS cells were stimulated with TNF‐α (20 ng/mL) for 1 h, followed by 24 h of drug containing serum treatment. Cells were resuspended in serum‐free medium (5 × 10⁵ cells/mL) and 200 µL of the suspension was added to the upper chamber. The lower chamber contained 500 µL of medium with 10% FBS as a chemoattractant. After 24 h, migratory cells were fixed with 4% paraformaldehyde for 20 min, stained with 0.1% crystal violet for 20 min, five fields were randomly selected and the average number of cells was calculated by image J software.

The scratch wound‐healing assay evaluated the lateral migration of RA‐FLS cells. RA‐FLS cells were seeded in 6‐well plates (4.5 × 10⁵/well), when cells reached > 95% confluence, they were serum free starved for 12 h to reduce proliferation effects. Scratches were created three scratches using a pipette tip to form nine intersections with pre‐drawn 3 lines, and detached cells were washed away with PBS. The cells were then treated with TNF‐α (20 ng/mL) and different drug containing serums. Images were taken at 0, 12, and 24 h under a microscope (Olympus IX‐8, Tokyo, Japan), and scratch areas were analyzed using Image J software.

#### 2.5.5 Cells Apoptosis of Hochest 33342 Staining and Mitochondrial Membrane Potential (MMP) Detection

Apoptosis was observed by hochest 33342 staining. RA‐FLS cells (2 × 10^5^ cells/well) were seeded in 6‐well plates, incubated with TNF‐α (20 ng/mL) for 1 h and drug containing serums for 24 h after cell adhesion. Each well was supplemented with 1 mL of hochest 33342 staining solution after removing the medium and incubated at 37°C for 30 min. After the incubation, the staining solution was discarded and the cells were washed three times with PBS. Subsequently, fluorescence detection was performed to observe the status of nuclear fragmentation.

MMP is also a useful indicator for early apoptosis detection. RA‐FLS cells (1 × 10⁵ cells/well) were treated with TNF‐α and drug containing serum as described previously. After incubation, the medium was removed and cells were washed with PBS. Fresh culture medium (1 mL) and JC‐1 staining working solution (1 mL) were added, followed by incubation at 37°C. Cells were then washed twice with JC‐1 staining buffer, and apoptosis was observed under a fluorescence microscope.

### 2.6 In Vivo Study on the Anti‐RA Mechanism of DBD

#### 2.6.1 Establishment of a Collagen‐Induced Arthritis (CIA) Rat Model and DBD Treatment

After 1 week of acclimatization feeding, 48 male wistar rats (SPF, 180 ± 20 g) were used. Randomization was performed using a random number generator to assign animals to different groups, ensuring unbiased distribution. Efforts were made to conduct the experiments in a single‐blind manner where feasible to minimize observer bias, and 40 were randomly selected for the establishment of the CIA model. Bovine type II collagen emulsion was prepared by mixing 10 mg of collagen powder with 5 mL acetic acid, allowing it to stand overnight, then vortexing with an equal volume of complete Freund's adjuvant. 40 rats were injected with 0.15 mL of the emulsion into the foot for the initial immunization, followed by a 0.10 mL booster injection at the same site 1 week later. 2 weeks after the initial immunization, rats were randomly divided to 6 groups (*n* = 8 per group): Normal (non‐modeling), model, positive (Tripterygium glycosides), DBD low‐dose (3.75 g/kg, DBD‐L), DBD middle‐dose (7.50 g/kg, DBD‐M), DBD high‐dose (15.0 g/kg, DBD‐H). The normal and model groups were administered distilled water, while the treatment groups were given either the positive drug or different doses of DBD. After 28 days of continuous intragastric administration, serum, spleen, thymus, and ankle joints were collected according to the for subsequent analysis. Arthritis severity was assessed based on the following criteria: (0) no swelling or erythema; (1) local toe joint affected; (2) local toe joint and dorsum pedis affected; (3) entire paw affected (including ankle); (4) maximal erythema, swelling, and motor dysfunction. The total score for all 4 limbs, with a maximum score of 16, was used to assess arthritis severity.

#### 2.6.2 Measurement of Inflammatory Factors in Serum

According to the relevant instructions, ELISA kits were used to measure pro‐inflammatory cytokines, including TNF‐α, IL‐1β, and IL‐6.

#### 2.6.3 Hematoxylin‐Eosin (HE) Staining

The pathological changes in the rat ankle joints were assessed using HE staining. The preserved joints were removed from the 4% paraformaldehyde solution and decalcified with EDTA for an appropriate duration. Following gradient dewaxing with dimethylbenzene and dehydration with ethanol, the tissues were embedded in paraffin. Slices about 5 μM thick were prepared and baked at 60°C for 1 h, followed by HE staining. The pathological changes of joints were observed under NIKON Ci‐S optical microscope (Tokyo, Japan). Additionally, the histopathological score was assessed based on a previously reported reference [21], primarily evaluating parameters such as inflammatory cell infiltration and pannus formation, etc.

#### 2.6.4 Micro‐CT Scanning Examination

Micro‐CT scanning was carried out to acquire the 3D reconstruction images of the CIA rats using a PerkinElmer Quantum GX micro CT system (Norwalk, CT, USA) with the PerkinElmer Analyze software (Version 12.0, Norwalk, CT, USA). Briefly, the preserved ankle joint tissue fixed by 4% paraformaldehyde was scanned by micro‐CT with the following scanning parameters, X‐ray: 90 kv and 88 μA, field of view (FOV): 72 mm, acquisition: 72, recon: 36, voxel Size: 72 μM, scan mode: High resolution, scan time: 4 min.

#### 2.6.5 Metabolomics Analysis

##### 2.6.5.1 Serum Sample Preparation

Serum samples were thawed at 4°C, and subsequently mixed with an equal volume of pre‐cooled methanol/acetonitrile/water (2:2:1, v/v). The mixture was vortexed and subjected to low‐temperature ultrasonication for 30 min. Then centrifuged at 14,000 g for 20 min at 4°C. The supernatant was collected and vacuum‐dried. The residue was then reconstituted in 100 μL acetonitrile/water (1:1, v/v), vortexed, and centrifuged again at 14,000 g for 15 min at 4°C. The final supernatant was used for further analysis.

##### 2.6.5.2 UHPLC‐Q‐Exactive Orbitrap MS/MS Analysis

The analysis was conducted using an UHPLC (Vanquish UHPLC, Thermo) system with a Waters ACQUITY UPLC BEH Amide column (1.7 µM, 2.1 mm × 100 mm) coupled with a Orbitrap (Q Exactive HF‐X/Q Exactive HF) in Shanghai Personal Biotechnology Co. Ltd. The mobile phase consisted of 25 mM ammonium acetate and 25 mM ammonium hydroxide in water (Phase A) and acetonitrile (Phase B). The gradient was 98% B for 1.5 min and was linearly reduced to 2% in 10.5 min, and then kept for 2 min, and then increased to 98% in 0.1 min, with a 3 min re‐equilibration period employed. Column temperature: 25°C, volume flow rate: 0.25 mL/min; injection volumn: 2 μL.

Both ESI positive and negative ion modes were used. The ESl source conditions were set as follows: Ion source gas1 (Gas1): 60, ion source gas2 (Gas2): 60, curtain gas (CUR): 30, source temperature: 600°C, IonSpray Voltage Floating (ISVF): ± 5500 V. In MS and auto MS/MS acquisition, the instrument was set to acquire over the m/z range 80–1200 Da and 70–1200 Da, respectively.

##### 2.6.5.3 Data Analysis

Raw MS data were converted to MzXML files using ProteoWizard MSConvert. Peak detection, alignment, and grouping were performed with XCMS, generating tables with m/z ratios, retention times, and peak intensities for each sample. Metabolite identification was based on accurate m/z values and MS/MS spectra, compared with an in‐house database of authentic standards. After sum normalization and pareto scaling, multidimensional statistical analysis data of metabolomics were analyzed using the R package (ropls), including multivariate analysis techniques such as PCA and orthogonal partial least‐squares discriminant analysis (OPLS‐DA). Model robustness was assessed via sevenfold cross‐validation and permutation testing. Variable importance in projection (VIP) values from the OPLS‐DA model were calculated to determine the contribution of each variable to classification. Statistical significance was evaluated using a student's t‐test, with metabolites identified as significant if VIP > 1 and *p* < 0.05, and MetaboAnalyst 5.0 was used for differential metabolite pathway analysis.

### 2.7 Western Blot Analysis

RA‐FLS cells (5 × 10^5^ cells/well) were stimulated and treated according to the previous method. Total protein from RA‐FLS cells and synovial tissue was extracted using RIPA lysis buffer containing 1% protease inhibitor and phosphatase inhibitor, and the protein concentration was measured using a BCA protein quantification kit. Total protein was separated by 10% sodium dodecyl sulfate polyacrylamide gel electrophoresis (SDS‐PAGE) and transferred to a 0.22 μm Polyvinylidene fluoride (PVDF) membrane. The membrane was blocked with 5% skim milk for 1.5 h at room temperature and subsequently incubated overnight at 4°C with primary antibodies specific for β‐actin, IκBα, p‐IκBα, p65, p‐p65, IKKα, p‐IKKα and NLRP3. Following washing with PBS, the membranes were incubated with secondary antibodies at room temperature for 1 h. The images were quantified using ImageJ software. All experiments were repeated three times.

### 2.8 Real‐Time Polymerase Chain Reaction (RT‐PCR) Analysis

RA‐FLS cells (4.5 × 10⁵ cells/well) after stimulation and intervention, as well as synovial tissue that had been weighed and ground, were used to extract total RNA with the RNAsimple Total RNA Kit. Subsequently, cDNA was synthesized according to the instructions of the TRANS reverse transcription kit. Subsequently, a 50 μL reaction mixture was prepared by combining 2× Hieff PCR Master Mix, cDNA template, primers, and water. RT‐PCR amplification was then performed to obtain the Ct values. The relative expression of each gene was quantified and normalized with the 2^−ΔΔCt^ method. The primers used for the RT‐PCR analysis are shown in Table 1. All experiments were repeated three times.

**Table 1:** Sequences of primers used in RT‐PCR.

Genes	Sequences
IκBα	F:5′‐TGCACTTGGCCATCATCCAT‐3′
	R:5′‐TCTGTTGACATCAGCCCCAC‐3′
p65	F:5′‐GCATTCTGACCTTGCCTATC‐3′
	R:5′‐CCAGTCTCCGAGTGAAGC‐3′
IKKα	F:5′‐AGCGAGCAGATGACGTATGG‐3′
	R:5′‐ATGTTCTGCTGAAGTCGGGG‐3′
β‐actin	F:5′‐GTCGTACCACTGGCATTGTG‐3′
	R:5′‐TCTCAGCTGTGGTGGTGAAG‐3′

### 2.9 Statistical Analysis

Data are presented as mean ± standard deviation (SD). One‐way analysis of variance (ANOVA) was applied to determine the statistical significance between the results of the control group and the model group, the model group and the treatment groups. *p* < 0.05 was considered statistically significant. All data were analyzed using Prism 9.5 (GraphPad Software, San Diego, CA, United States).

## 3 Results

### 3.1 Bioinformatics Analysis

#### 3.1.1 Gene Expression Data Merging and DEGs Screening of RA

**Figure 2:**
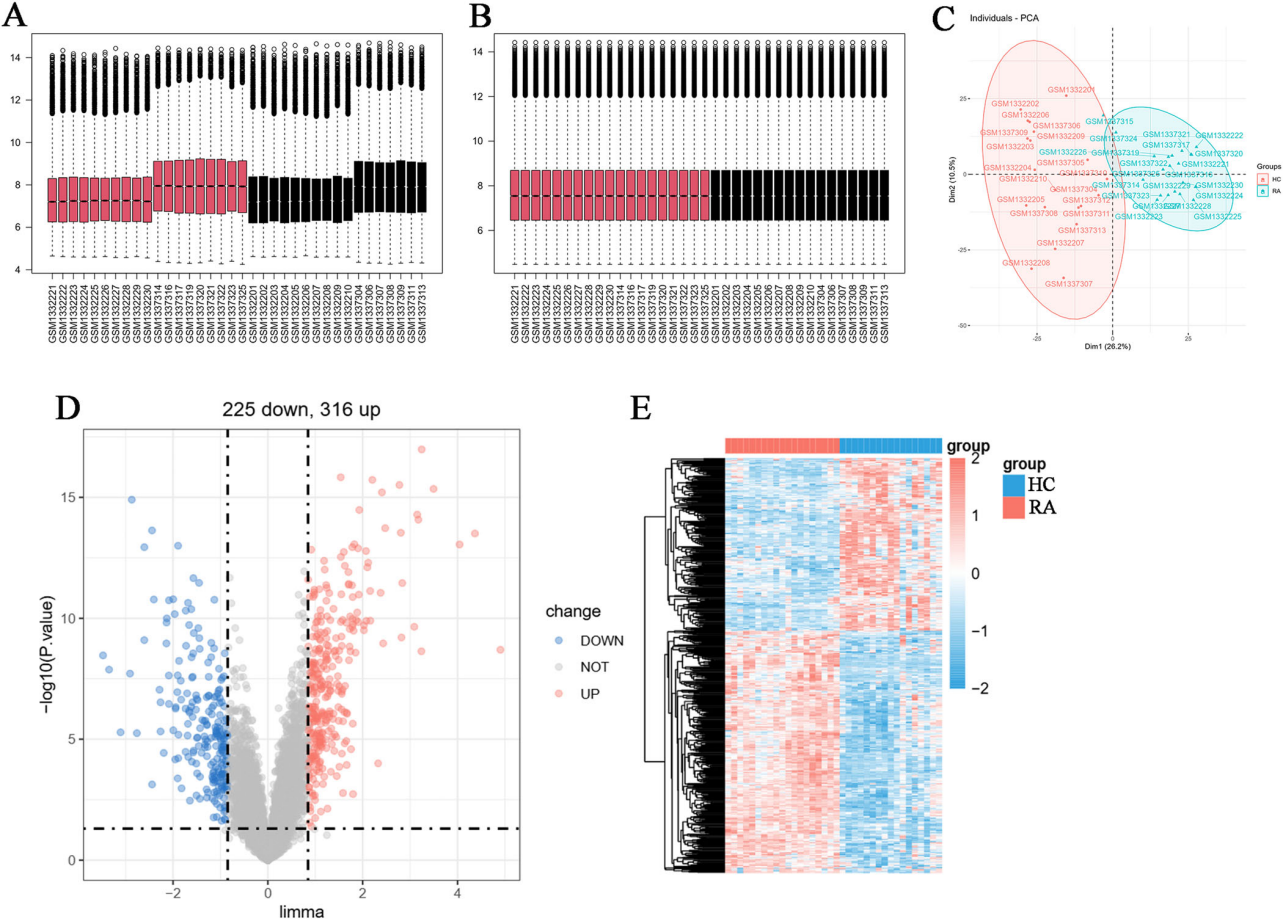
The processing results of unified data set of GSE55457 and GSE55235 datasets. (A, B) Box plots before and after batch effect removal. (C) Principal component analysis (PCA) analysis of gene expression patterns between healthy control (HC) group and rheumatoid arthritis (RA) group. (D, E) Volcano plots and heatmaps illustrate the differential epression genes (DEGs).

Batch effects between the two datasets were corrected, and expression levels were normalized, allowing for their integration into a unified data set (Figure 2A,B). PCA revealed a clear separation in gene expression patterns between the healthy control (HC) group and the RA patient group (Figure 2C). DEGs analysis screened 316 significantly upregulated genes and 225 significantly downregulated genes (|logFC | ≥ 1, *p* < 0.05), which were visualized using volcano plots and heatmaps (Figure 2D,E).

#### 3.1.2 Enrichment Analysis of DEGs

**Figure 3:**
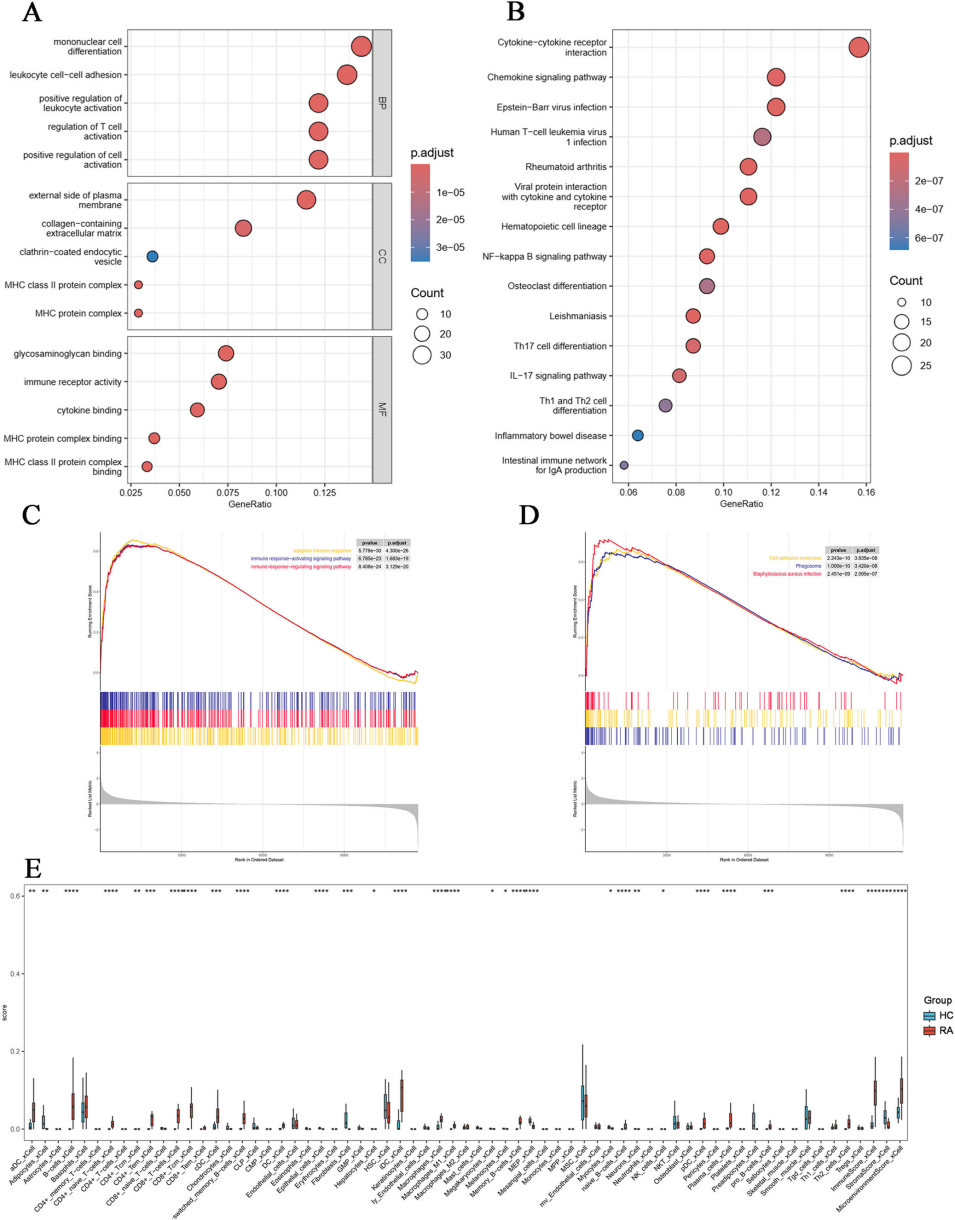
GO, KEGG and GSEA enrichment analysis results of DEGs. (A, B) Bubble plot of GO and KEGG analysis result. (C, D) GSEA‐GO and GSEA‐KEGG enrichment analysis curve. (E) Immune cell infiltration analysis of DEGs between HC group and RA group.

The results of GO analysis (Figure 3A) showed that positive regulation of leukocyte activation and regulation of T cell activation were the main biological processes (BP). In the cellular component (CC), the DEGs were mainly distributed in the external side of the plasma membrane and collagen‐containing extracellular matrix. In molecular function (MF), the functions related to glycosaminoglycan binding and immune receptor activity were significantly enriched. KEGG analysis (Figure 3B) showed that DEGs were significantly enriched in chemokine signaling pathway, NF‐κB signaling pathway and IL‐17 signaling pathway, suggesting that DEGs play an important role in inflammatory response and immune regulation. In addition, the GSE55457 and GSE55235 datasets in GSEA were used to confirm the close association between RA and adaptive immune response, immune response activation signaling pathway, cell adhesion molecule and phagosome (Figure 3C,D).

#### 3.1.3 Analysis of Immune Cell Infiltration

Immune cell infiltration analysis (Figure 3E) showed that there were significant differences in 32 immune cells between the healthy control (HC) group and the RA group (*p* < 0.05). The results showed that the levels of CD8 + T cells, memory B cells, B cells and others were significantly upregulated in the synovial tissue of RA patients, while the levels of adipocytes, fibroblasts, NKT cells and others were significantly downregulated.

#### 3.1.4 Targets Screening and Components Identification of DBD

**Figure 4:**
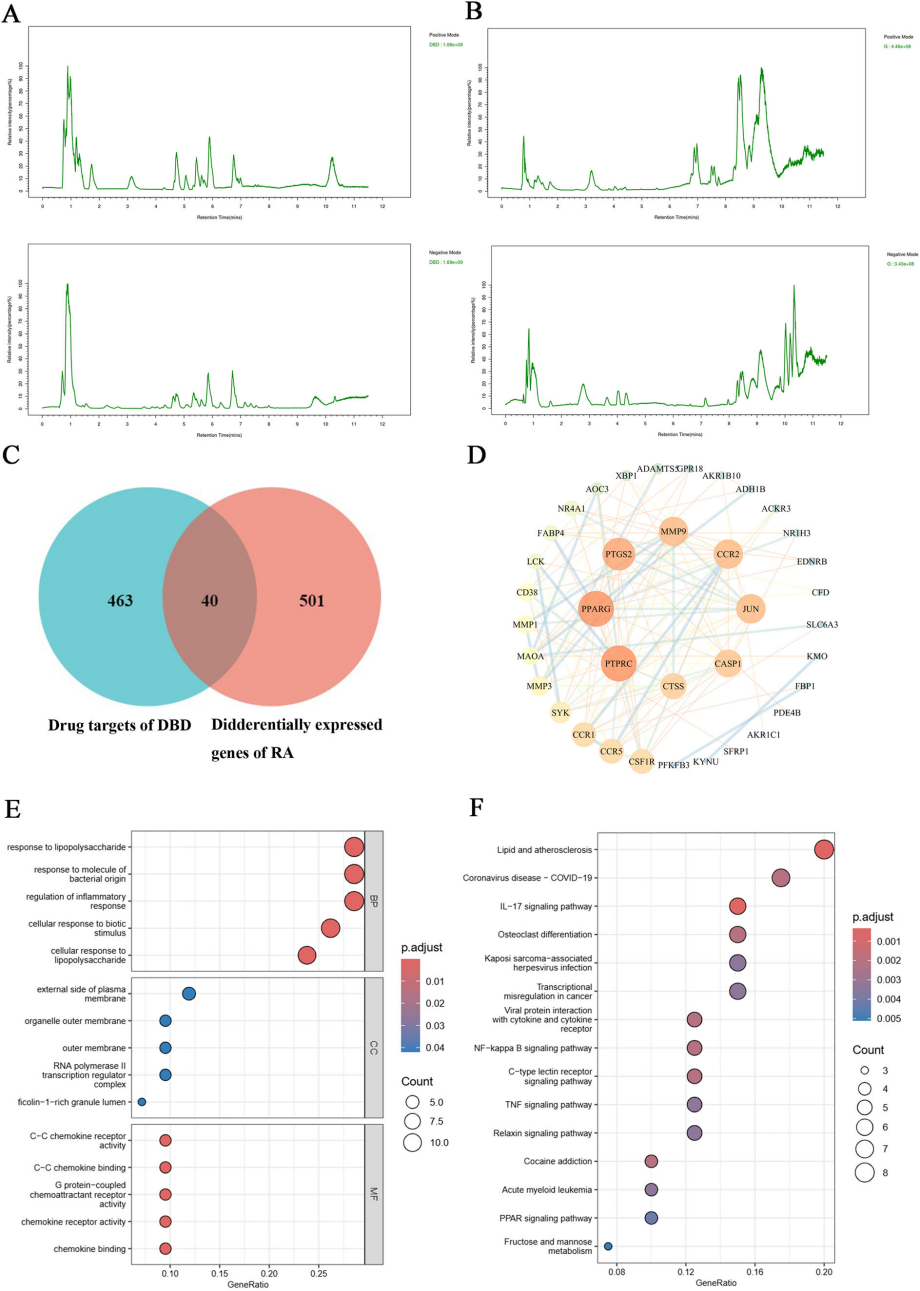
Comprehensive analysis of the components and pharmacological mechanisms of danggui buxue decoction (DBD) in anti‐RA. (A, B) Base peak chromatogram (BPC) of chemical components and blood‐absorbed components of DBD in both positive and negative ion modes. (C) Venn diagram of 40 hub genes between DBD drug targets and RA‐related DEGs. (D) Protein‐protein interaction (PPI) network of the 40 hub genes. (E, F) GO and KEGG enrichment analysis of the 40 hub genes.

The chemical and blood‐absorbed components of DBD were systematically identified. Chemical profiling revealed 83 components using UPLC‐MS/MS in both positive and negative ionization modes (ppm < 5), as shown in the base peak chromatogram (BPC) in Figure 4A (Table S1). After excluding endogenous substances present in the blank serum, 34 blood‐absorbed components were identified under the same analytical conditions (ppm < 5), with the corresponding BPC presented in Figure 4B (Table S2). Based on these 34 blood‐absorbed components, a total of 503 potential therapeutic targets of DBD were predicted following duplicate removal.

#### 3.1.5 Identification of Hub Genes and Construction of PPI Network

The intersecting genes between DBD targets and DEGs were identified as hub genes critical for the anti‐RA effects of DBD (Figure 4C). In total, 40 hub genes were identified in this analysis. These hub genes were subsequently uploaded to the STRING database to construct a PPI network (Figure 4D). The top 10 hub genes were listed in Table 2 based on their degree values.

**Table 2:** Top 10 genes ranked by PPI analysis results.

Gene nzme	Average shortest path length	Betweenness centrality	Closeness centrality	Degree
PTPRC	1.722222	0.098511	0.580645	18
PPARG	1.611111	0.236528	0.62069	18
PTGS2	1.638889	0.186721	0.610169	16
CCR2	1.861111	0.048846	0.537313	14
MMP9	1.833333	0.039949	0.545455	14
JUN	1.916667	0.037939	0.521739	14
CASP1	1.888889	0.032053	0.529412	13
CTSS	1.944444	0.019741	0.514286	12
CCR1	1.916667	0.123895	0.521739	11
CCR5	2.027778	0.048758	0.493151	11

#### 3.1.6 Enrichment Analysis of Hub Genes

GO and KEGG analyses were conducted on the 40 hub genes. GO analysis revealed the functional distribution of these genes across the three categories: BP, CC, and MF (Figure 4E). KEGG enrichment analysis showed that drug targets were significantly enriched in inflammation‐related pathways, including IL‐17, NF‐κB, and TNF signaling pathways. Given its role as a classic pro‐inflammatory pathway central to various inflammation‐related diseases [22], and based on KEGG analysis of DEGs in RA, the NF‐κB signaling pathway was selected as the focus for further research and validation (Figure 4F).

### 3.2 Results of In Vitro Study of DBD on RA

#### 3.2.1 DBD Inhibited the Proliferation of RA‐FLS Cells

**Figure 5:**
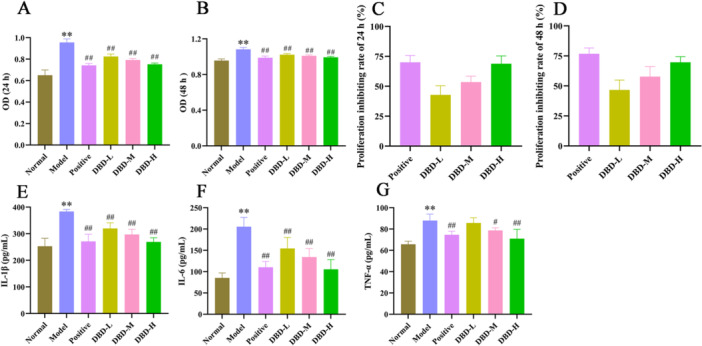
DBD inhibited the proliferation and the releases of inflammatory cytokines of human rheumatoid arthritis fibroblast‐like synoviocyte (RA‐FLS) cells. (A, B) The OD values of 24 and 48 h of cell counting kit‐8 (CCK‐8) assay. (C, D) The proliferation inhibiting rates of 24 and 48 h of CCK‐8 assay. (E–G) Effects of DBD on cytokines of IL‐1β, IL‐6, and TNF‐α in RA‐FLS cell culture supernatant. The data were presented as the means ± SD and ANOVA was applied to determine the statistical significance, the significant differences were indicated as *p < 0.05, **p < 0.01 versus normal group. #*p* < 0.05, ##*p* < 0.01 versus model group.

As shown in Figure 5A and B, OD values in the model group were significantly higher than in the normal group (*p* < 0.01). DBD drug containing serum reduced OD values in a dose‐dependent manner (*p* < 0.01), similar to the positive group. Quantitative analysis confirmed that DBD inhibited TNF‐α‐stimulated RA‐FLS proliferation in a concentration‐ and time‐dependent manner, with the strongest effect observed in the DBD‐H group, comparable to the positive (Figure 5C,D).

#### 3.2.2 DBD Inhibited the Releases of Inflammatory Cytokines

The anti‐inflammatory effects of DBD drug containing serum were assessed by measuring inflammatory factors in the cell culture supernatant. As shown in Figure 5E–G, IL‐1β, IL‐6, and TNF‐α levels were significantly increased in the model group compared to the normal group (*p* < 0.01), treatment with drug containing serum significantly reduced the levels of these inflammatory factors. These results demonstrate that DBD effectively inhibits the inflammatory response.

#### 3.2.3 DBD Inhibited the Migration of RA‐FLS Cells

**Figure 6:**
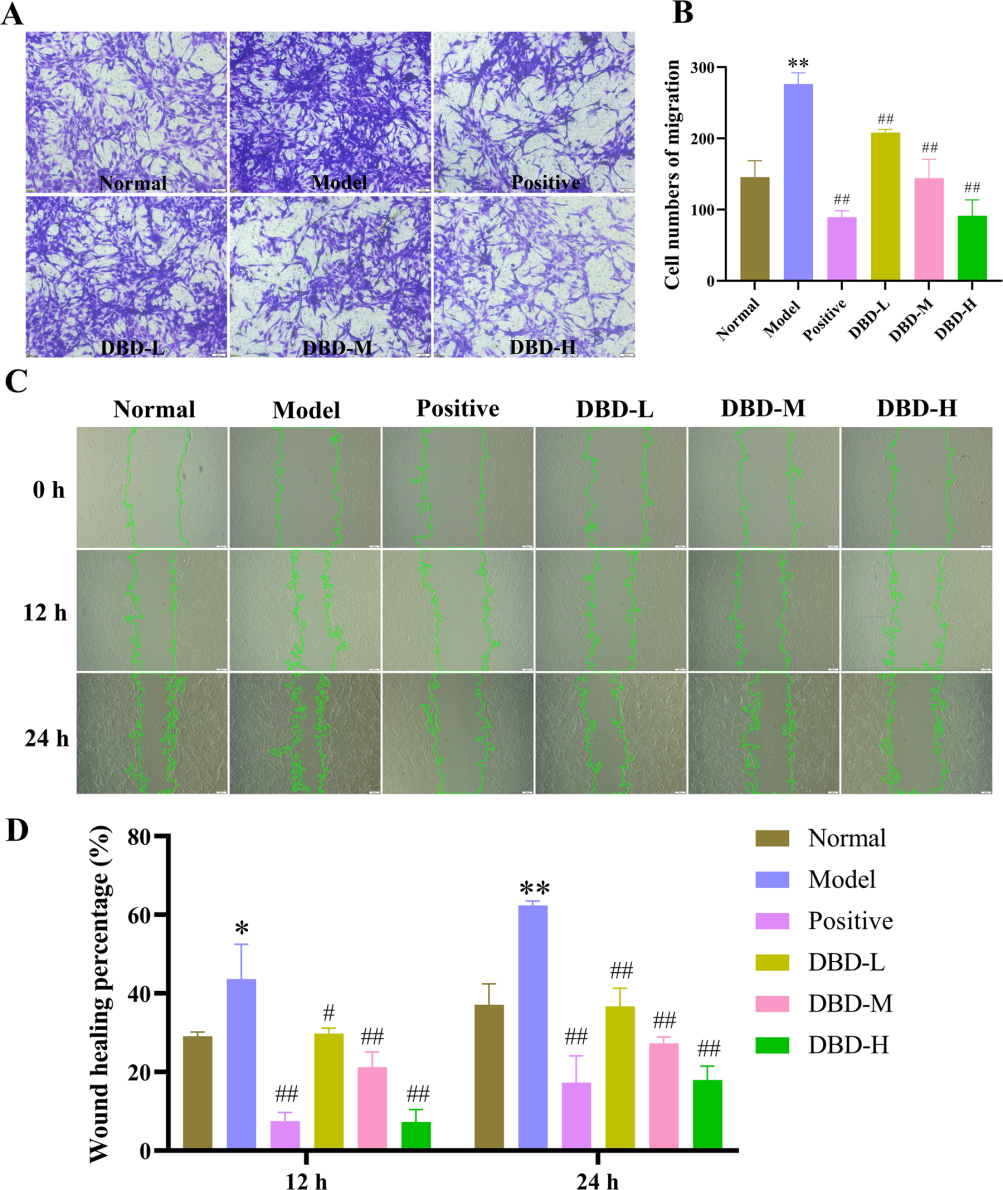
DBD inhibited the migration of RA‐FLS. (A) Transwell assay images of migrated cells, scale bar: 200 μm (40× magnification). (B) Cell counts from five randomly selected fields. (C, D) Three random fields were photographed at 0, 12, and 24 h, scale bar: 200 μM (40× magnification). Results of scratch areas were expressed as the percentage reduction in initial scratch width at each time point. The data were presented as the means ± SD and ANOVA was applied to determine the statistical significance, the significant differences were indicated as *p < 0.05, **p < 0.01 versus normal group. #*p* < 0.05, ##*p* < 0.01 versus model group.

The migration ability of RA‐FLS cells was assessed. TNF‐α stimulation significantly enhanced the number of RA‐FLS cells and their ability to migrate across the permeable membrane (Figure 6A and B). Treatment with DBD drug containing serum significantly reduced TNF‐α‐induced cell migration in a dose‐dependent manner (*p* < 0.01). These findings were further validated by wound healing assays (Figure 6C,D).

#### 3.2.4 DBD Promoted Apoptosis of RA‐FLS Cells

**Figure 7:**
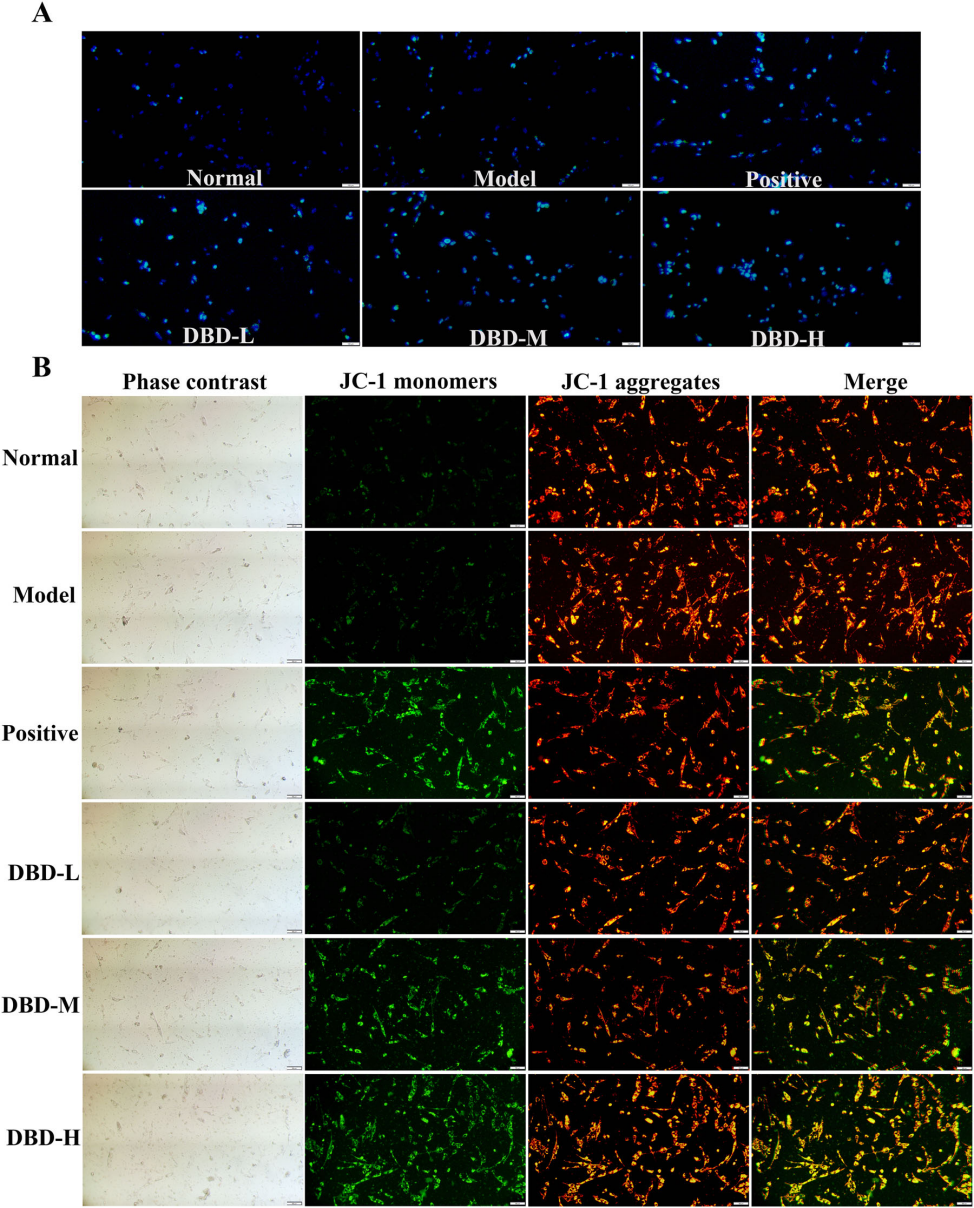
Apoptosis‐inducing effect of DBD on RA‐FLS cells. (A) Apoptotic assay by hochest 33342 staining, scale bar: 100 μM (100× magnification). (B) Apoptotic assay by mitochondrial membrane potential (MMP), scale bar: 50 μM (200× magnification).

Apoptosis was assessed using hoechst 33342 staining and MMP analysis. In apoptotic cells, nuclear staining is enhanced, fluorescence appears brighter, and nuclear fragmentation occurs. In the normal and model groups, cells exhibited weaker fluorescence intensity and less fragmentation after hoechst staining compared to the positive group and the DBD drug containing serum treatment groups, indicating that the positive group and the DBD treatment group had the ability to induce apoptosis of RA‐FLS cells (Figure 7A). Figure 7B illustrates the changes in mitochondrial membrane potential, inferred through JC‐1 staining, which showed green fluorescence in the mitochondria of both groups. In contrast, the mitochondria of the positive drug and DBD‐treated groups exhibited a shift from red to green fluorescence, resulting in a significant decrease in the red‐to‐green fluorescence intensity ratio. This suggests a loss of MMP associated with the early stages of apoptosis. Additionally, the DBD drug containing serum treatment groups showed varying degrees of apoptosis, with the DBD‐H group displaying the most pronounced apoptotic features.

### 3.3 Results of In Vivo Study of DBD on RA

#### 3.3.1 DBD Reduced the Inflammatory Response

**Figure 8:**
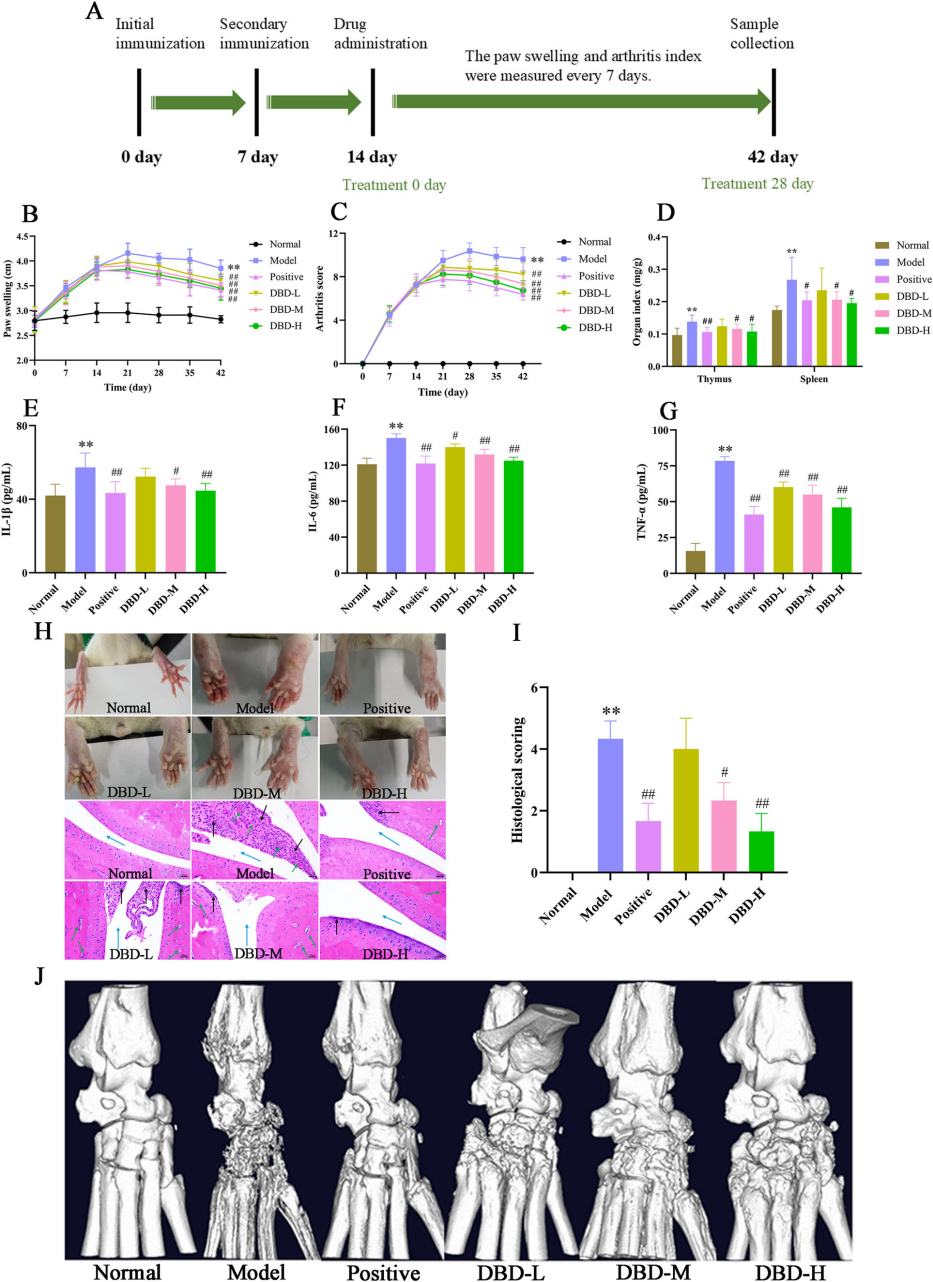
Anti‐RA effects of DBD on collagen‐induced arthritis (CIA) rats. (A) Experimental procedure for evaluating the anti‐RA effects of DBD in rats. (B–D) DBD's effects on paw swelling, arthritis score and organ index in CIA rats. (E–G) DBD's effects on serum levels of pro‐inflammatory cytokines IL‐1β, IL‐6, and TNF‐α. (H) The represented rat paws in different groups and histological sections of the ankle joints stained with hematoxylin‐eosin (HE). (I) Histological scoring of HE. (J) Bone destruction of the ankle joints scanning with Micro‐CT. Blue arrows indicate the articular cavity, black arrows indicate inflammatory cell infiltration and green arrows point to pannus formation. The data were presented as the means ± SD and ANOVA was applied to determine the statistical significance, the significant differences were indicated as *p < 0.05, **p < 0.01 versus normal group. #*p* < 0.05, ##*p* < 0.01 versus model group.

The experimental procedure is summarized in Figure 8A. The experimental procedure is summarized in Figure 8A. As shown in Figure 8B,C, the model group exhibited significant RA symptoms compared with the normal group, including marked paw swelling and arthritis scores. Following 4 weeks of treatment, the DBD groups showed a significant reduction in both paw swelling (*p* < 0.01) and arthritis scores (*p* < 0.01) compared to the model group. A similar trend was observed in the positive group. The immune organ index (Spleen and thymus) are presented in Figure 8D. The model group exhibited significantly higher spleen and thymus indices than the normal group (*p* < 0.01). Treatment with different DBD doses reduced these indices compared to the model group, with the DBD‐H group having the most pronounced effect (*p* < 0.05). A comparable reduction was also seen in the positive group (*p* < 0.05). As shown in Figure 8E–G, the results demonstrated that these pro‐inflammatory cytokines of serum were significantly higher in the model group compared to normal group (*p* < 0.01), confirming the successful establishment of the CIA rat model. The serum levels of IL‐1β, IL‐6 and TNF‐α in the positive group and the various DBD dose groups were significantly reduced, indicating that DBD effectively inhibits the inflammatory response, with the DBD‐H showing the best effect (*p* < 0.01).

#### 3.3.2 DBD Inhibited the Inflammatory Reactions and Reduced the Bone Destruction of Ankle Joint of CIA Rats

Inflammatory responses and bone destruction in the ankle joint were assessed using HE staining and Micro‐CT. At the end of the experiment, visible changes were observed in the paws of rats across different treatment groups (Figure 8H). The model group exhibited significant swelling and redness, while the positive and DBD treatment groups showed varying degrees of improvement. HE staining results showed that a large number of inflammatory cells infiltrated and pannus formed in the model group. These pathological changes were notably alleviated in the positive, DBD‐M, and DBD‐H groups, indicating a protective effect (Figure 8H). Figure 8I showed the histological scoring of HE staining. Micro‐CT scanning showed that rough bone surfaces and severe erosive changes in the model group (Figure 8J). These conditions were markedly changed following treatment with positive group and various doses of DBD.

#### 3.3.3 DBD Changed the Serum Metabolite Profile of CIA Rats

##### 3.3.3.1 Multivariate Statistical Analysis

QC samples were used to evaluate the reproducibility and accuracy of metabolomics results. The results showed that the analysis system was stable and the data were reliable (Figure S1A–D).

**Figure 9:**
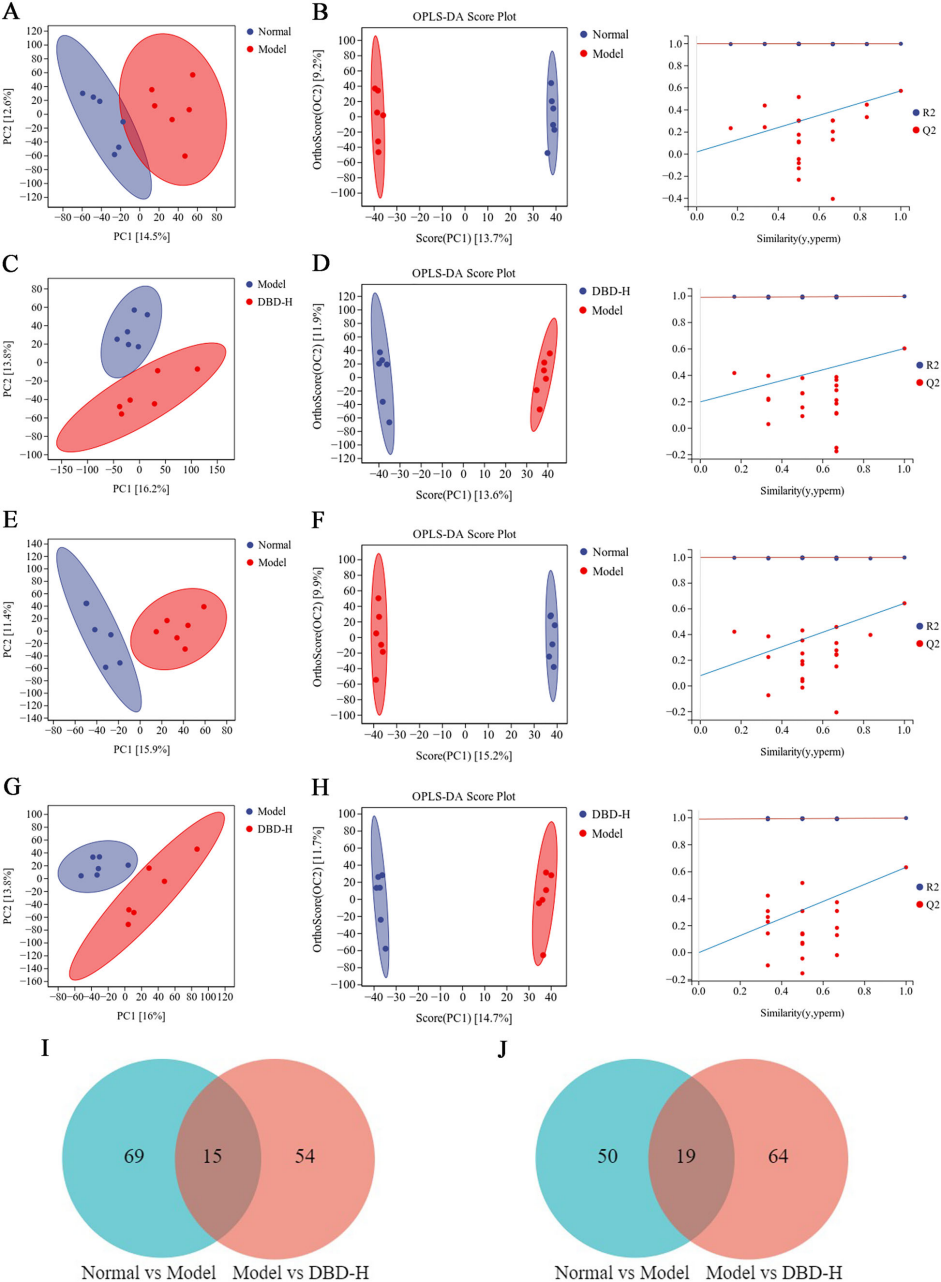
Multivariate statistical analysis of serum metabolites based on UHPLC‐Q‐Exactive Orbitrap MS/MS data acquired in both positive and negative ion modes. (A–D) Normal versus model group and Model versus DBD‐H group in positive ion mode of PCA and orthogonal partial least‐squares discriminant analysis (OPLS‐DA) score plots. (E–H) Normal versus model group and model versus DBD‐H group in negative ion mode of PCA and OPLS‐DA score plots. (I, J) The venn diagram of differential metabolites of the identified significantly in both the model group versus the normal group and the model group versus the DBD‐H group in positive and negative ion mode.

**Figure 10:**
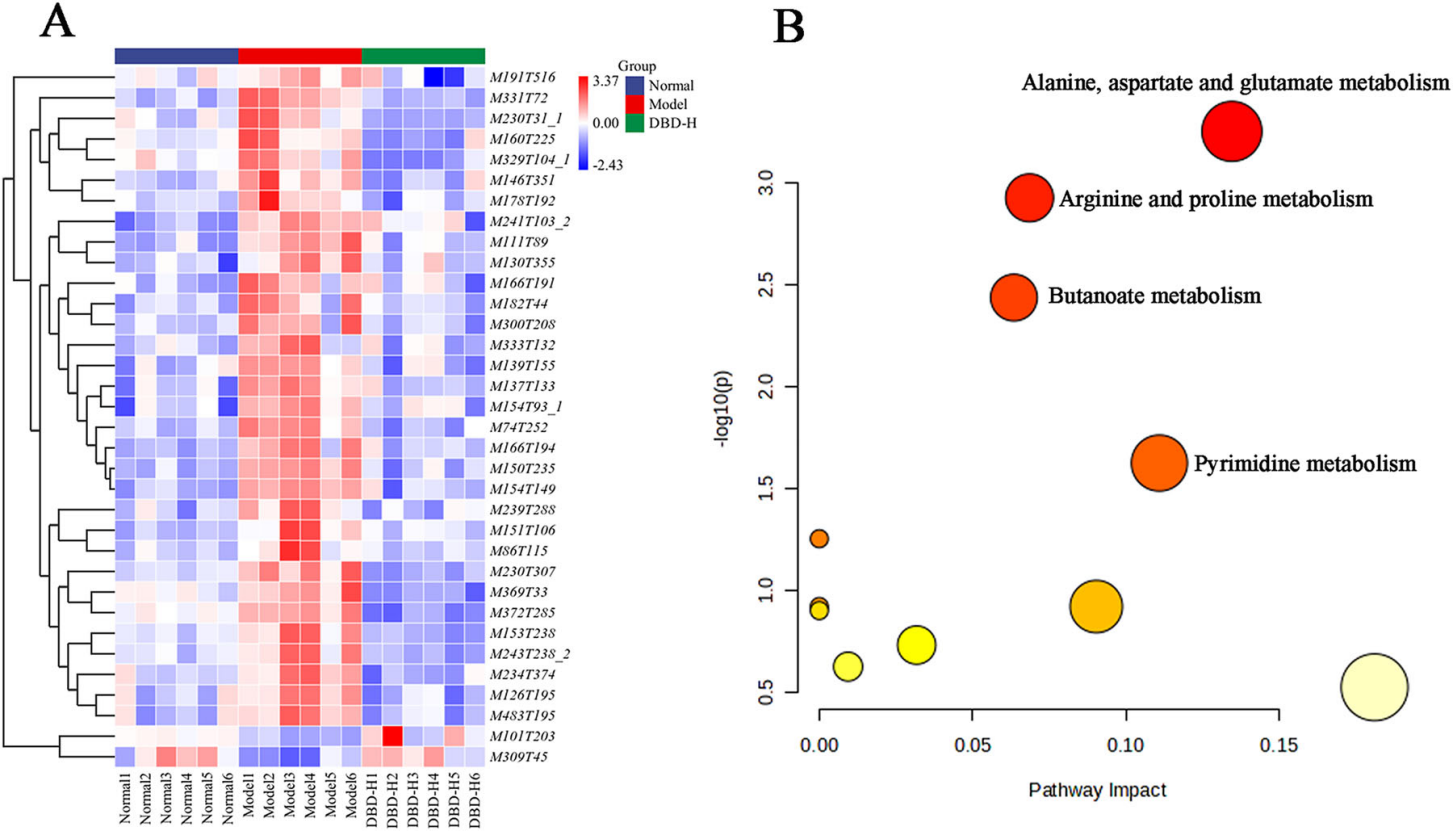
Results of metabolomics. (A) The dynamic trends of the differential metabolites. (Red indicates an upregulation of metabolites and blue indicates a downregulation of metabolites. (B) Metabolic pathway analysis of 34 differential metabolites in serum samples (MetaboAnalyst 5.0).

Metabolomic data analysis involves using multivariate statistical methods, such as PCA and OPLS‐DA, to reduce dimensionality and perform regression on multi‐dimensional data while preserving as much original information as possible. These methods help identify metabolite differences across groups [23]. PCA results reveal clear distinctions between samples from different groups in both positive and negative ion modes, highlighting significant metabolic differences (Figure 9A,C,E,G). OPLS‐DA, an extension of PCA, offers better discriminative power, especially with large within‐group differences [24]. OPLS‐DA analysis was applied to identify differential metabolites associated with CIA‐induced RA (Figure 9B, D, F, H). The venn diagram (Figure 9I,J) illustrates ions identified in both the model versus normal and model versus DBD‐H groups, representing differential metabolites (VIP > 1, *p* < 0.05). This approach screened out 15 and 19 endogenous differential metabolites in positive and negative ion modes, respectively, as potential efficacy‐related biomarkers for assessing DBD intervention effects in CIA rats. Detailed information is provided in Table 3. The dynamic trends of differential metabolites across the normal, model, and DBD‐H groups are shown in Figure 10A.

**Table 3:** Identification results of 34 differential metabolites in serum by UHPLC‐Q‐Exactive Orbitrap MS/MS.

No.	ID	Metabolites	m/z	RT (s)	Formula	HMDB	CAS	Ion mode	Identification confidence	Normal vs Model			Model vs DBD‐H		
										Fold change	p value	Trends	Fold change	p value	Trends
1	M154T93_1	Fludarabine	154.0496	92.815	C_10_H_12_N_5_O_4_	—	21679‐14‐1	Positive	0.8880	2.5039	0.0015	up	0.5447	0.0036	down
2	M151T106	D‐xylose	151.0610	105.943	C_5_H_10_O_5_	HMDB0060254	58‐86‐6	Positive	0.9998	2.1883	0.0100	up	0.5741	0.0347	down
3	M86T115	Gamma.‐aminobutyric acid	86.0601	115.349	C_4_H_9_NO_2_	HMDB0000112	56‐12‐2	Positive	0.9570	1.5633	0.0366	up	0.6506	0.0021	down
4	M154T149	N‐acetylhistamine	154.0974	149.291	C_7_H_11_N_3_O	HMDB0013253	673‐49‐4	Positive	0.9957	2.4783	0.0000	up	0.4756	0.0398	down
5	M139T155	Salicylic acid	139.0502	154.559	C_7_H_6_O_3_	HMDB0001895	69‐72‐7	Positive	0.7980	1.9367	0.0026	up	0.4465	0.0021	down
6	M166T194	7‐methylguanine	166.0721	193.766	C_6_H_7_N_5_O	HMDB0000897	578‐76‐7	Positive	0.9977	1.5373	0.0009	up	0.7102	0.0055	down
7	M483T195	5‐methyl‐2’‐deoxycytidine	483.2200	194.806	C_10_H_15_N_3_O_4_	—	7838/7/3	Positive	0.9934	1.7161	0.0053	up	0.5135	0.0010	down
8	M126T195	5‐methylcytosine	126.0663	195.065	C_5_H_7_N_3_O	HMDB0002894	554‐01‐8	Positive	0.9999	1.3365	0.0088	up	0.6601	0.0011	down
9	M160T225	Dl‐2‐aminocaprylic acid	160.1332	224.636	C_8_H_17_NO_2_	HMDB0000991	644‐90‐6	Positive	0.9910	1.8313	0.0261	up	0.2743	0.0051	down
10	M150T235	3‐methyladenine	150.0766	235.434	C_6_H_7_N_5_	HMDB0011600	5142‐23‐4	Positive	0.9986	1.8364	0.0000	up	0.5725	0.0001	down
11	M74T252	N,n‐dimethylformamide	74.0713	251.685	C_3_H_7_NO	HMDB0001888	1968/12/2	Positive	0.9412	1.9970	0.0002	up	0.4422	0.0002	down
12	M372T285	Tamoxifen	372.2381	285.099	C_26_H_29_NO	HMDB0014813	10540‐29‐1	Positive	0.8910	1.6046	0.0005	up	0.2248	0.0000	down
13	M239T288	5‐hydroxyflavone	239.1002	287.7235	C_15_H_10_O_3_	HMDB0141534	491‐78‐1	Positive	0.9865	1.4521	0.0120	up	0.6772	0.0111	down
14	M146T351	G‐guanidinobutyrate	146.0922	350.682	C_5_H_11_N_3_O_2_	HMDB0003464	463‐00‐3	Positive	0.9996	2.3383	0.0033	up	0.3689	0.0047	down
15	M234T374	2’,3’‐dideoxycytidine	234.0825	373.538	C_9_H_13_N_3_O_3_	HMDB0015078	7481‐89‐2	Positive	0.9473	1.6997	0.0038	up	0.4491	0.0009	down
16	M230T31_1	Acetaminophen sulfate	230.0125	30.653	C_8_H_9_NO_5_S	HMDB0059911	32113‐41‐0	Negative	0.9692	1.9067	0.0282	up	0.2609	0.0014	down
17	M369T33	Mollicellin i	369.1379	33.037	C_21_H_22_O_6_	HMDB0139292	1016605‐29‐0	Negative	0.9977	1.7108	0.0113	up	0.2071	0.0001	down
18	M178T192	Trans‐ferulic acid	362.2372	33.789	C_10_H_10_O_4_	HMDB0000954	537‐98‐4	Negative	0.9966	1.7322	0.0140	up	0.4979	0.0106	down
19	M182T44	4‐pyridoxic acid	182.0461	43.920	C_9_H_11_NO_3_	HMDB0000017	82‐82‐6	Negative	0.9912	2.8416	0.0106	up	0.3920	0.0139	down
20	M309T45	Eicosenoic acid	309.2801	45.271	C_20_H_38_O_2_	—	5561‐99‐9	Negative	0.9888	0.6979	0.0069	down	1.4175	0.0033	up
21	M331T72	Prostaglandin e3	331.1917	72.460	C_20_H_30_O_5_	HMDB0002664	802‐31‐3	Negative	0.9748	3.6647	0.0002	up	0.2250	0.0001	down
22	M111T89	Uracil	111.0200	88.810	C_4_H_4_N_2_O_2_	HMDB0000300	66‐22‐8	Negative	0.9998	1.6305	0.0002	up	0.6902	0.0014	down
23	M241T103_2	His‐ser	241.0831	103.137	C_9_H_14_N_4_O_4_	HMDB0000273	21438‐60‐8	Negative	0.9721	1.4231	0.0000	up	0.8381	0.0250	down
24	M329T104_1	Aurantio‐obtusin	329.0702	103.909	C_17_H_14_O_7_	—	67979‐25‐3	Negative	0.9800	1.5147	0.0391	up	0.2545	0.0005	down
25	M333T132	Prostaglandin a2	333.2073	132.098	C_20_H_30_O_4_	HMDB0002752	13345‐50‐1	Negative	0.9965	1.7417	0.0230	up	0.6004	0.0468	down
26	M137T133	3,4‐dihydroxybenzaldehyde	137.0357	133.193	C_7_H_4_O_3_	HMDB0059965	139‐85‐5	Negative	0.9860	2.3755	0.0016	up	0.4776	0.0015	down
27	M166T191	Guanidinoethyl sulfonate	166.018	190.753	C_3_H_9_N_3_O_3_S	HMDB0003584	543‐18‐0	Negative	0.9965	1.3267	0.0045	up	0.8010	0.0319	down
28	M101T203	Succinic semialdehyde	101.0244	203.4475	C_4_H_6_O_3_	HMDB0001259	692‐29‐5	Negative	0.8766	0.6132	0.0000	down	1.6124	0.0456	up
29	M300T208	N‐Acetyl‐d‐Glucosamine 6‐Phosphate	300.0397	208.261	C_8_H_16_NO_9_P	HMDB0001367	3616‐42‐0	Negative	0.9794	1.6515	0.0070	up	0.5771	0.0063	down
30	M153T238	O,o‐diethyl phosphate	153.0298	237.548	C_4_H_11_O_2_PS_2_	HMDB0012209	762‐04‐9	Negative	0.9626	1.2987	0.0074	up	0.6815	0.0010	down
31	M243T238_2	Pseudouridine	243.0623	237.554	C_9_H_12_N_2_O_6_	HMDB0000767	1445‐07‐4	Negative	0.9980	1.2996	0.0100	up	0.6885	0.0015	down
32	M230T307	Imazapic	230.1147	306.992	C_14_H_17_N_3_O_3_	—	104098‐48‐8	Negative	0.8088	1.7005	0.0013	up	0.4417	0.0002	down
33	M130T355	Hydroxyproline	130.051	355.4935	C_5_H_9_NO_3_	HMDB0000725	51‐35‐4	Negative	0.9973	1.8442	0.0057	up	0.6456	0.0201	down
34	M191T516	Citrate	191.0199	515.81	C_6_H_5_O_7_	HMDB0000094	77‐92‐9	Negative	0.9899	1.2616	0.0240	up	0.6122	0.0250	down

*Note:* Student's *t*‐test was applied to determine the statistical significance, with metabolites identified as significant if VIP > 1 and *p* < 0.05.

##### 3.3.3.2 Metabolic Pathways Analysis

To further investigate the mechanisms underlying the anti‐RA effects of DBD, 34 differential metabolites identified were subjected to pathway analysis using pathway analysis module of MetaboAnalyst 5.0. The analysis revealed that 11 metabolic pathways were perturbed. Among them, 4 pathways showed significant regulatory effects (*p* < 0.05), including alanine, aspartate and glutamate metabolism, arginine and proline metabolism, butanoate metabolism and pyrimidine metabolism, as can be seen in Figure 10B, with the related data given in Table 4.

**Table 4:** Detailed results from the metabolic pathway analysis (MetaboAnalyst 5.0).

Pathway metabolism	Total	Expected	Hits	Raw p	Log10(p)	Holm adjust
Alanine, aspartate and glutamate metabolism	28	0.1778	3	0.0006	3.2548	0.0445
Arginine and proline metabolism	36	0.2286	3	0.0012	2.9281	0.0932
Butanoate metabolism	15	0.0952	2	0.0036	2.4380	0.2845
Pyrimidine metabolism	39	0.2476	2	0.0237	1.6248	1
Vitamin B6 metabolism	9	0.0571	1	0.0559	1.2530	1
Pantothenate and CoA biosynthesis	20	0.1270	1	0.1203	0.9198	1
Citrate cycle (TCA cycle)	20	0.1270	1	0.1203	0.9198	1
beta‐Alanine metabolism	21	0.1333	1	0.1260	0.8998	1
Glyoxylate and dicarboxylate metabolism	32	0.2032	1	0.1861	0.7304	1
Amino sugar and nucleotide sugar metabolism	42	0.2667	1	0.2374	0.6245	1
Drug metabolism ‐ cytochrome P450	55	0.3492	1	0.2999	0.5231	1

#### 3.3.4 DBD Suppressed the Activation of the NF‐κB of RA‐FLS Cells and Synovial Tissue

The NF‐κB signaling pathway plays a critical role in regulating physiological processes such as immune response, inflammation, cell growth, and apoptosis. To investigate the anti‐inflammatory mechanism of DBD, this study systematically analyzed the key proteins and mRNA levels of the NF‐κB signaling pathway in RA‐FLS cells and synovial tissue of rats across the normal, model, positive, and DBD‐H groups, building on previous research findings.

**Figure 11:**
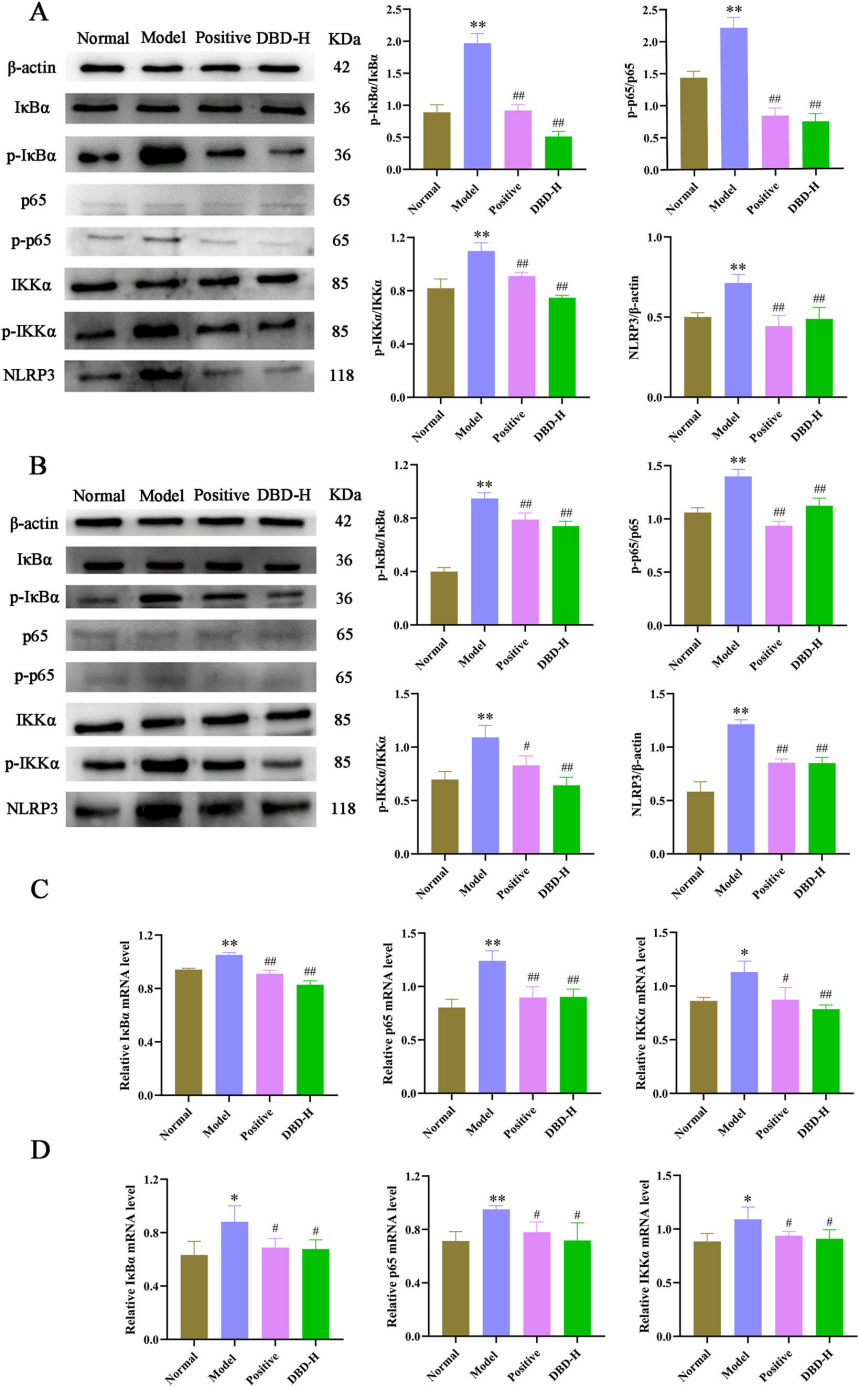
Effect of DBD on the expression of NF‐κB signaling pathway‐related proteins and mRNA levels. (A, B) Expression of p‐ IκB/IκB, p‐p65/p65, p‐IKKα/IKKα and NLRP3 proteins in the RA‐FLS cells and synovial tissue of rat. (C, D) Relative mRNA levels of IκBα, p65, and IKKα in the RA‐FLS cells and synovial tissue of rat. The data were presented as the means ± SD and ANOVA was applied to determine the statistical significance, the significant differences were indicated as *p < 0.05, **p < 0.01 versus normal group. #*p* < 0.05, ##*p* < 0.01 versus model group.

Western blot results showed that the expression levels of p‐IκBα, p‐p65, and p‐IKKα were significantly increased in the model group in both RA‐FLS cells and synovial tissue (*p* < 0.01), accompanied by the activation of NLRP3 (*p* < 0.01). However, DBD‐H treatment effectively suppressed these phosphorylation levels and NLRP3 expression (*p* < 0.01), with inhibitory effects comparable to those of the positive group. Meanwhile, the total protein levels of IκBα, p65, and IKKα showed no significant changes (*p* > 0.05) (Figure 11A,B). Furthermore, RT‐PCR results revealed that the mRNA expression levels of IκBα, p65, and IKKα mRNA were significantly increaseed in the model group (*p* < 0.05), while DBD‐H treatment markedly downregulated these gene expressions (*p* < 0.05) (Figure 11C,D).

## 4 Discussion

DBD has shown therapeutic potential in various autoimmune diseases, including RA. Previous studies indicate that DBD can inhibit inflammation and oxidative stress, regulate autophagy and enhance immune function through multi‐target pathways [13]. To systematically elucidate the mechanism of action of DBD, this study employed serum pharmacochemistry combined with UPLC‐MS/MS to identify 83 chemical components and 34 blood‐absorbed components in DBD, establishing a preliminary material basis for its pharmacological effects. Bioinformatics analysis highlighted the NF‐κB signaling pathway as a critical role for its anti‐inflammatory activity, with further validation confirming its role in DBD's therapeutic mechanism for RA. Metabolomics also highlighted the significant therapeutic potential of DBD in alleviating RA by modulating amino acid metabolism.

### 4.1 Key Genes and Inflammation

Previous studies have demonstrated that key regulatory genes‐including PTPRC, PPARG, PTGS2, and CCR2‐are critically involved in the pathogenesis of RA and the modulation of inflammatory responses. PTPRC (Also known as CD45) regulates immune cell activation and excessive immune responses [25], while PPARG suppresses pro‐inflammatory factors and inhibits RA‐FLS cells proliferation [26]. PTGS2 (Also known as COX‐2) promotes prostaglandin synthesis, exacerbating inflammation and joint damage [27]. CCR2 plays a key role in the immune inflammatory response of RA by mediating the chemotaxis of macrophages to inflammatory sites. The signal axis composed of CCR2 and ligand CCL2 is an important mechanism to promote synovial inflammation and joint destruction [28, 29]. These genes collectively drive RA‐related inflammation and tissue damage, making them promising therapeutic targets. In this study, based on the PPI network constructed from the intersection of DBD‐related targets and DEGs in RA, we identified 40 hub genes and ranked them according to their degree centrality. PTPRC, PPARG, PTGS2 and CCR2 emerged as top‐ranking nodes in the network, suggesting their pivotal roles in the molecular mechanism underlying DBD's therapeutic effects against RA. This finding is highly consistent with previous studies and further supports the role of these genes in the regulation of RA‐related inflammation and therapeutic intervention.

### 4.2 RA‐FLS Cells and Inflammation

RA‐FLS are the key drivers of the pathological mechanisms in RA. Under normal conditions, FLS maintain synovial homeostasis by synthesizing synovial fluid and essential components of articular cartilage. However, previous evidence indicates that in the pathological microenvironment of RA, FLS undergo a significant phenotypic transformation, shifting from joint homeostasis‐maintaining “protectors” to key drivers of inflammation and tissue destruction [30]. Nygaard and Wu have demonstrated that this phenotypic transformation endows FLS with enhanced proliferative and migratory capacities. Activated RA‐FLS secrete large amounts of pro‐inflammatory cytokines, such as IL‐1β, IL‐6, and TNF‐α, along with proteolytic enzymes, which collectively contribute to synovial hyperplasia, cartilage degradation, and exacerbated bone resorption [31, 32]. In our study, following TNF‐α stimulation, RA‐FLS exhibited significantly increased proliferation and migratory ability, increased release of inflammatory cytokines, and reduced apoptosis. Notably, these pathological changes were reversed to varying degrees after DBD intervention, suggesting that DBD effectively modulates the aberrant activation of RA‐FLS, thereby suppressing their pro‐inflammatory and migratory behaviors and exerting anti‐inflammatory and joint‐protective effects. This finding not only validates previous conclusions regarding the critical role of RA‐FLS in disease progression, but also provides experimental evidence supporting the anti‐RA effects of DBD through regulation of RA‐FLS behavior, further elucidating its underlying therapeutic mechanisms.

### 4.3 NF‐κB Signaling Pathway and Inflammation

The NF‐κB signaling pathway serves as a central regulator of inflammatory responses and tissue damage in RA, with p65, IκBα, IKKα, and their phosphorylation states being critical for pathway activation. Previous studies have shown that under pathological conditions of RA, p‐p65 enhances NF‐κB activity, while p‐IκBα undergoes degradation, releasing NF‐κB to translocate into the nucleus and activate pro‐inflammatory gene expression. p‐IKKα acts as a key upstream event initiating this pathway, playing a direct role in modulating the phosphorylation and degradation of IκBα [33, 34]. To further investigate the role of the NF‐κB signaling pathway in the pathogenesis of RA, we employed both CIA rat models and TNF‐α‐stimulated RA‐FLS cell models. Our findings revealed that DBD intervention effectively blocked the excessive activation of the NF‐κB signaling pathway, primarily by significantly reducing the phosphorylation levels of key pathway proteins in synovial tissues and RA‐FLS cells, such as p‐IκBα, p‐p65, and p‐IKKα, as well as by inhibiting the overactivation of the NLRP3 inflammasome. Furthermore, DBD was found to downregulate the mRNA expression of p65, IκBα, and IKKα, suggesting that its inhibitory effects on the NF‐κB pathway occur not only at the posttranslational level but also involve transcriptional regulation. These findings are consistent with the studies by Jimi and Liu et al., who reported that sustained activation of NF‐κB not only promotes the overexpression of pro‐inflammatory cytokines such as TNF‐α, IL‐6, and IL‐1β, but also induces abnormal apoptosis in RA‐FLS cells. The accumulation of these apoptotic cells on the surfaces of articular cartilage and bone contributes to progressive joint destruction [35, 36].

TNF‐α, IL‐6, and IL‐1β are key pro‐inflammatory cytokines in the pathogenesis of RA, each playing distinct roles at different stages of the inflammatory response. TNF‐α acts as a central driver in RA, promoting the expression of IL‐6 and IL‐1β, which in turn contribute to synovial hyperplasia and joint destruction [37]. Both IL‐1β and TNF‐α upregulate matrix metalloproteinases (MMPs), accelerating cartilage degradation and leading to irreversible joint damage. A positive feedback loop exists between MMPs and IL‐1β, where IL‐1β induces MMPs, and MMPs activity further trigger the release of pro‐inflammatory cytokines, perpetuating a cycle of inflammation [38]. Additionally, TNF‐α and IL‐1β stimulate COX‐2 expression, which intensifies both inflammation and pain [39]. IL‐6, a critical mediator of chronic inflammation, sustains immune activation by stimulating B and T cells and inducing acute‐phase proteins, prolonging systemic inflammation. It also promotes Th17 cell differentiation, which enhances IL‐17 production [40]. IL‐17, in synergy with TNF‐α and IL‐1β, worsens synovial inflammation, accelerates cartilage degradation, and exacerbates tissue damage [41].

**Figure 12:**
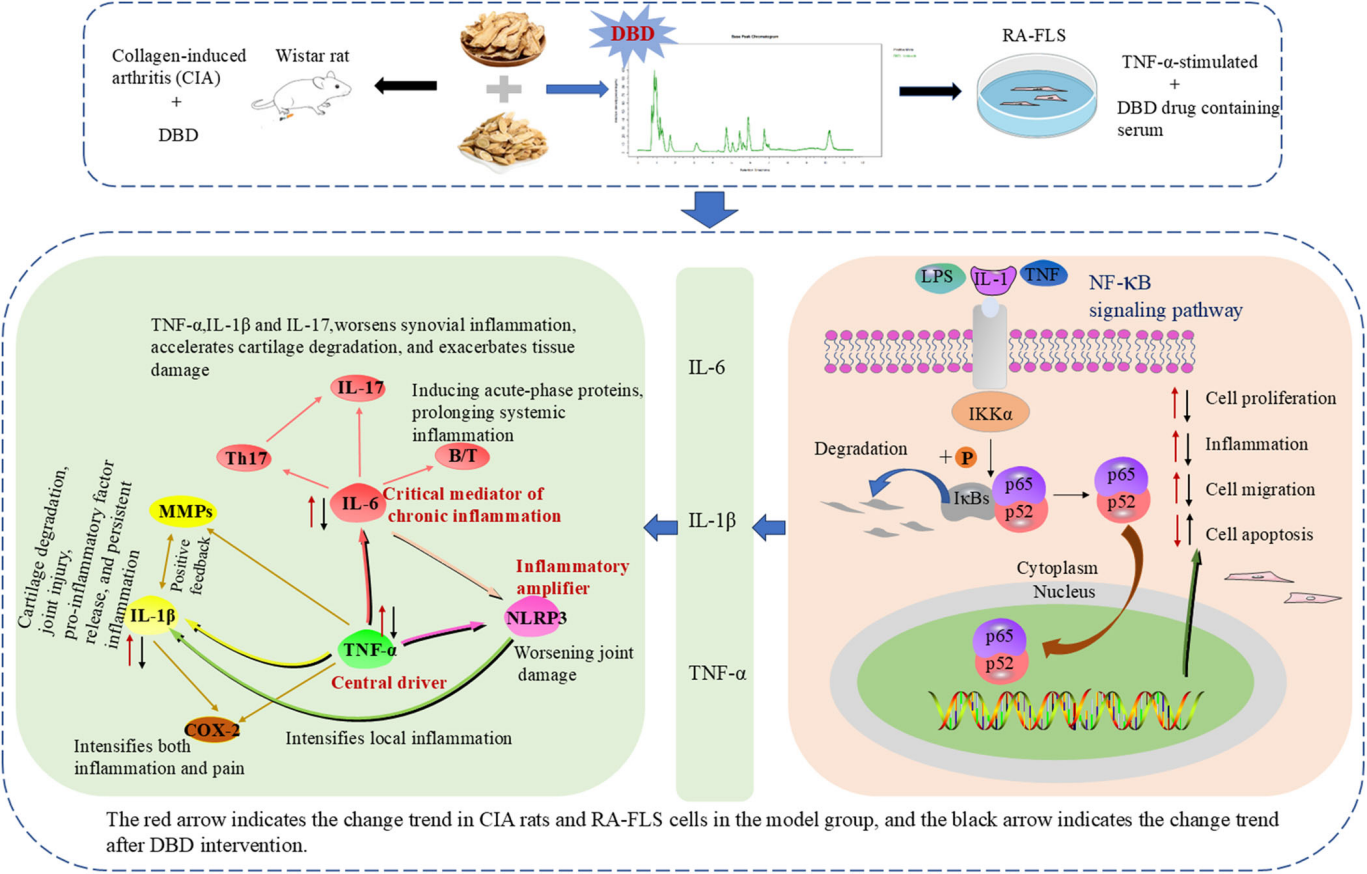
The possible schematic depiction. DBD inhibits RA‐FLS cell proliferation and migration, induces apoptosis, and suppresses cytokine release (TNF‐α, IL‐1β, IL‐6) and NF‐κB pathway activation.

At the same time, the interaction between the NF‐κB signaling pathway and the NLRP3 inflammasome plays a crucial role in the inflammatory mechanism of RA. Pro‐inflammatory factors TNF‐α and IL‐1β further activate the NF‐κB signaling pathway through positive feedback, while increasing the expression of NLRP3 and promoting the maturation and release of IL‐1β, thereby forming a vicious cycle that accelerates the progression of RA [42, 43]. The possible schematic depiction are summarized in Figure 12. This study further confirms the central role of the NF‐κB signaling pathway in the progression of RA and highlights DBD as a potential therapeutic drug for modulating this key inflammatory pathway. The findings not only provide pharmacological evidence for DBD's anti‐inflammatory and joint‐protective effects, but also align with its traditional functions in TCM, namely “clearing heat and detoxifying” and “activating blood circulation to resolve stasis”, thereby offering a solid scientific basis for its application in the treatment of RA.

### 4.4 Metabolic Pathways and Inflammation

The alanine, aspartate, and glutamate metabolism, arginine and proline metabolism, butanoate metabolism and pyrimidine metabolism pathways that are critical for energy production, immune regulation and inflammation control, which are often disrupted in RA. DBD treatment helps restore these metabolic pathways, highlighting its therapeutic potential for RA management. Alanine, aspartate, and glutamate metabolism supports amino acid synthesis and energy metabolism while also regulating T cell activation and cytokine release. Disruption of this pathway in CIA rats indicates imbalanced energy homeostasis and heightened inflammation [44]. Arginine and proline metabolism influences nitric oxide (NO) production, which regulates vascular tone and immune responses in RA. Excessive NO exacerbates inflammation and tissue damage, while proline, vital for collagen synthesis, supports tissue repair [45]. Butanoate metabolism, which involves the production of SCFAs like butyrate, plays a crucial role in maintaining intestinal barrier integrity and regulating immune responses. Impaired butyrate metabolism in RA reduces SCFA levels, leading to gut barrier dysfunction and systemic inflammation [46]. Alterations in the pyrimidine metabolism pathway can affect the production of inflammatory factors, DNA damage repair, and normal cell proliferation and differentiation [47]. DBD significantly regulates these four metabolic pathways, alleviating inflammation and minimizing joint damage, demonstrating comprehensive therapeutic effects in immune modulation, anti‐inflammation, and tissue repair, offering a new direction for the treatment of RA.

Studies have shown that inflammatory factors such as TNF‐α, IL‐6 and IL‐1β can interfere with amino acid metabolism by activating NF‐κB signaling pathway, especially alanine, aspartate and glutamate metabolism, as well as arginine and proline metabolism, leading to cell dysfunction and promoting the progression of chronic inflammatory diseases such as RA [48, 49]. In the present study, we hypothesize that DBD may restore the inflammation‐disrupted metabolic balance by inhibiting the release of the aforementioned pro‐inflammatory cytokines, thereby improving abnormal amino acid metabolism and alleviating metabolic disturbance caused by chronic inflammation. This mechanism may represent a novel integrative therapeutic strategy for diseases such as RA, offering not only anti‐inflammatory effects but also multi‐target regulation through modulation of metabolic pathways. These findings highlight the potential of DBD as a systemic regulator within the complex disease network.

This study has the following limitations: First, although the discovery of animal models provides valuable insights, interspecies differences limit their direct applicability to humans and therefore require further clinical validation. Second, only the inflammation‐related NF‐κB signaling pathway involving hub genes was experimentally validated, other signaling pathways associated with hub genes, as well as 4 key metabolic pathways related to differential metabolites, were not validated in this study.

## 5 Conclusion

In conclusion, this study is the first to comprehensively analyze the main targets and pathways of DBD in anti‐RA by combining bioinformatics, serum pharmacochemistry, in vivo and in vitro experiments, and metabolomics. We demonstrated that DBD inhibits the proliferation and migration of RA‐FLS cells, induces cell apoptosis, alleviates RA symptoms in CIA rats, and regulates the of alanine, aspartate and glutamate metabolism, arginine and proline metabolism, butyrate metabolism, and pyrimidine metabolism. The underlying mechanism of these effects may be related to the inhibition of NF‐κB signaling pathway and the reduction of inflammation, which may be mediated by the 34 blood‐absorption components of DBD. These findings suggest that DBD holds significant potential as a novel therapeutic drug for the prevention and anti‐RA effects. Future studies should aim to validate other signaling pathways associated with the hub genes, as well as metabolic pathways related to differential metabolites, to provide a more comprehensive and accurate elucidation of the underlying mechanisms. Furthermore, future research can focus on more in‐depth investigations, such as alterations in gut microbiota in CIA rats and the regulatory effects of DBD on the gut microbiota.

## Author Contributions

**Lianyun Du:** methodology, validation, writing – original draft. **Ye Zhai:** writing – original draft, validation, investigation. **Meixiu Luo:** software, validation, investigation. **Lu Tang:** software, supervision. **Saiyue Qiu:** methodology. **Xin Jiang:** methodology. **Enpeng Wang:** writing – review and editing, project administration. **Zhi Pan:** writing – review and editing, project administration, funding acquisition.

## Ethics Statement

All animal experimental protocol adheres to the guidelines for international animal experiments approved by the Animal Management and Use Committee of Changchun University of Chinese Medicine (No. 2025365).

## Conflicts of Interest

The authors declare no conflicts of interest.

## Supporting information

Supplementary materials.

Author Checklist Full.

## Data Availability

Research data are not shared. The datasets used and/or analyzed during the current study are available from the corresponding author upon reasonable request.

## References

[1] S. Liu , J. Li , and L. Feng , “Gallic Acid Regulates Immune Response in a Mouse Model of Rheumatoid Arthritis,” Immunity, Inflammation and Disease 11, no. 2 (2023): e782. 36840490 10.1002/iid3.782 PMC9933205

[2] Q. Zhang , W. Peng , S. Wei , et al., “Guizhi‐Shaoyao‐Zhimu Decoction Possesses Anti‐Arthritic Effects on Type II Collagen‐Induced Arthritis in Rats via Suppression of Inflammatory Reactions, Inhibition of Invasion & Migration and Induction of Apoptosis in Synovial Fibroblasts,” Biomedicine & Pharmacotherapy = Biomedecine & pharmacotherapie 118 (2019): 109367–109381. 31545276 10.1016/j.biopha.2019.109367

[3] J. Cai , L. C. Zhang , R. J. Zhao , et al., “Chelerythrine Ameliorates Rheumatoid Arthritis by Modulating the AMPK/mTOR/ULK‐1 Signaling Pathway,” Phytomedicine 104 (2022): 154140‐154140. 35752081 10.1016/j.phymed.2022.154140

[4] X. Ba , Y. Huang , P. Shen , et al., “WTD Attenuating Rheumatoid Arthritis *via* Suppressing Angiogenesis and Modulating the PI3K/AKT/mTOR/HIF‐1α Pathway,” Frontiers in Pharmacology 12 (2021): 696802–696818. 34646130 10.3389/fphar.2021.696802 PMC8502817

[5] L. Yao , S. Cheng , J. Yang , et al., “Metabolomics Reveals the Intervention Effect of Zhuang Medicine Longzuantongbi Granules on a Collagen‐Induced Arthritis Rat Model by Using UPLC‐MS/MS,” Journal of Ethnopharmacology 294 (2022): 115325. 35508204 10.1016/j.jep.2022.115325

[6] Y. Han , J. Wang , M. Jin , L. Jia , C. Yan , and Y. Wang , “Shentong Zhuyu Decoction Inhibits Inflammatory Response, Migration, and Invasion and Promotes Apoptosis of Rheumatoid Arthritis Fibroblast‐Like Synoviocytes via the MAPK p38/PPAR*γ*/CTGF Pathway,” BioMed Research International 2021 (2021): 6187695. 33511203 10.1155/2021/6187695 PMC7826240

[7] L. H. Calabrese and S. Rose‐John , “IL‐6 Biology: Implications for Clinical Targeting in Rheumatic Disease,” Nature Reviews Rheumatology 10, no. 12 (2014): 720–727. 25136784 10.1038/nrrheum.2014.127

[8] X. Li and S. Zhang , “Herbal Compounds for Rheumatoid Arthritis: Literatures Review and Cheminformatics Prediction,” Phytotherapy Research 34, no. 1 (2020): 51–66. 31515874 10.1002/ptr.6509

[9] L. Shi , Y. Zhao , C. Feng , et al., “Therapeutic Effects of Shaogan Fuzi Decoction in Rheumatoid Arthritis: Network Pharmacology and Experimental Validation,” Frontiers in Pharmacology 13 (2022): 967164–967180. 36059943 10.3389/fphar.2022.967164 PMC9428562

[10] S. Lü , Q. Wang , G. Li , S. Sun , Y. Guo , and H. Kuang , “The Treatment of Rheumatoid Arthritis Using Chinese Medicinal Plants: From Pharmacology to Potential Molecular Mechanisms,” Journal of Ethnopharmacology 176 (2015): 177–206. 26471289 10.1016/j.jep.2015.10.010

[11] X. Miao , S. Li , B. Xiao , J. Yang , and R. Huang , “Metabolomics Study of the Effect of Danggui Buxue Tang on Rats With Chronic Fatigue Syndrome,” Biomedical Chromatography 36, no. 7 (2022): e5379–e5389. 35373377 10.1002/bmc.5379

[12] P. Wang , Z. Wang , Z. Zhang , et al., “A Review of the Botany, Phytochemistry, Traditional Uses, Pharmacology, Toxicology, and Quality Control of the *Astragalus memeranaceus* ,” Frontiers in Pharmacology 14 (2023): 1242318. 37680711 10.3389/fphar.2023.1242318 PMC10482111

[13] C. Ma , Y. Jiang , Y. Wang , and R. Xu , “The Latest Research Advances of Danggui Buxue Tang as an Effective Prescription for Various Diseases: A Comprehensive Review,” Current Medical Science 42, no. 5 (2022): 913–924. 36245031 10.1007/s11596-022-2642-0

[14] L. Sun , Z. Yang , W. Zhao , et al., “Integrated Lipidomics, Transcriptomics and Network Pharmacology Analysis to Reveal the Mechanisms of Danggui Buxue Decoction in the Treatment of Diabetic Nephropathy in Type 2 Diabetes Mellitus,” Journal of Ethnopharmacology 283 (2022): 114699–114708. 34610419 10.1016/j.jep.2021.114699

[15] D. Li , J. Si , Y. Guo , et al., “Danggui‐Buxue Decoction Alleviated Vascular Senescence in Mice Exposed to Chronic Intermittent Hypoxia Through Activating the NRF2/HO‐1 Pathway,” Pharmaceutical Biology 61, no. 1 (2023): 1041–1053. 37431236 10.1080/13880209.2023.2230753 PMC10337509

[16] Z. Li , J. Yuan , Y. Dai , and Y. Xia , “Integration of Serum Pharmacochemistry and Metabolomics to Reveal the Underlying Mechanism of Shaoyao‐Gancao‐Fuzi Decoction to Ameliorate Rheumatoid Arthritis,” Journal of Ethnopharmacology 326 (2024): 117910. 38373664 10.1016/j.jep.2024.117910

[17] Y. Liu , H. Chen , G. Yang , and F. Feng , “Metabolomics and Serum Pharmacochemistry Combined With Network Pharmacology Uncover the Potential Effective Ingredients and Mechanisms of Yin‐Chen‐Si‐Ni Decoction Treating ANIT‐Induced Cholestatic Liver Injury,” Journal of Ethnopharmacology 335 (2024): 118713. 39163894 10.1016/j.jep.2024.118713

[18] Z. He , Z. Liu , and L. Gong , “Biomarker Identification and Pathway Analysis of Rheumatoid Arthritis Based on Metabolomics in Combination With Ingenuity Pathway Analysis,” Proteomics 21, no. 11/12 (2021): e2100037–e2100063. 33969925 10.1002/pmic.202100037

[19] K. W. Cheng , J. Shi , C. Huang , et al., “Integrated Metabolomics and Serum‐Feces Pharmacochemistry‐Based Network Pharmacology to Reveal the Mechanisms of an Herbal Prescription Against Ulcerative Colitis,” Computers in Biology and Medicine 178 (2024): 108775. 38941901 10.1016/j.compbiomed.2024.108775

[20] X. Zhang , P. Dong , J. Song , et al., “Identification and Mechanism Prediction of Mulberroside A Metabolites In Vivo and In Vitro of Rats Using an Integrated Strategy of UHPLC‐Q‐Exactive Plus Orbitrap MS and Network Pharmacology,” Frontiers in Chemistry 10 (2022): 981173–981196. 36238092 10.3389/fchem.2022.981173 PMC9552072

[21] K. Gan , L. Xu , X. Feng , et al., “Celastrol Attenuates Bone Erosion In Collagen‐Induced Arthritis Mice and Inhibits Osteoclast Differentiation and Function in RANKL‐Induced RAW264.7,” International Immunopharmacology 24, no. 2 (2015): 239–246. 25529994 10.1016/j.intimp.2014.12.012

[22] T. Lawrence , “The Nuclear Factor NF‐B Pathway in Inflammation,” Cold Spring Harbor Perspectives in Biology 1, no. 6 (2009): a001651. 20457564 10.1101/cshperspect.a001651 PMC2882124

[23] D. Yu , K. Zhang , J. Wu , X. Li , G. Zhou , and Y. Wan , “Integrated Metabolomic and Transcriptomic Analysis Revealed the Flavonoid Biosynthesis and Regulation in *Areca catechu* ,” Phytochemical Analysis 34, no. 3 (2023): 372–380. 36813748 10.1002/pca.3216

[24] L. Y. Du , H. E. Zhang , Y. Zhang , et al., “Comparative Study on Chemical Constituents of Ginseng Flowers With Four Consecutive Cultivation Age,” International Journal of Analytical Chemistry 2023 (2023): 1–12. 10.1155/2023/1771563 PMC10089779 37057128

[25] J. An , P. Chen , X. Li , X. Li , and F. Peng , “Identification of Potential Hub Genes and Biological Mechanism in Rheumatoid Arthritis and Non‐Small Cell Lung Cancer via Integrated Bioinformatics Analysis,” Translational Oncology 45 (2024): 101964. 38657441 10.1016/j.tranon.2024.101964 PMC11059132

[26] Q. Geng , J. Xu , X. Cao , et al., “Pparg‐Mediated Autophagy Activation Alleviates Inflammation in Rheumatoid Arthritis,” Journal of Autoimmunity 146 (2024): 103214. 38648706 10.1016/j.jaut.2024.103214

[27] W. Wang , S. Zhai , W. Yang , et al., “Acacetin Alleviates Rheumatoid Arthritis by Targeting HSP90 ATPase Domain to Promote COX‐2 Degradation,” Phytomedicine 135 (2024): 156171. 39489991 10.1016/j.phymed.2024.156171

[28] L. A. Tylaska , L. Boring , W. Weng , et al., “Ccr2 Regulates the Level of MCP‐1/CCL2 In Vitro and At Inflammatory Sites and Controls T Cell Activation in Response to Alloantigen,” Cytokine 18, no. 4 (2002): 184–190. 12126640 10.1006/cyto.2002.1031

[29] D. Flegar , M. Filipović , A. Šućur , et al., “Preventive CCL2/CCR2 Axis Blockade Suppresses Osteoclast Activity in a Mouse Model of Rheumatoid Arthritis by Reducing Homing of CCR2^hi^ Osteoclast Progenitors to the Affected Bone,” Frontiers in immunology 12 (2021): 767231. 34925336 10.3389/fimmu.2021.767231 PMC8677701

[30] V. Tsaltskan and G. S. Firestein , “Targeting Fibroblast‐Like Synoviocytes in Rheumatoid Arthritis,” Current Opinion in Pharmacology 67 (2022): 102304–102311. 36228471 10.1016/j.coph.2022.102304 PMC9942784

[31] G. Nygaard and G. S. Firestein , “Restoring Synovial Homeostasis in Rheumatoid Arthritis by Targeting Fibroblast‐Like Synoviocytes,” Nature Reviews Rheumatology 16, no. 6 (2020): 316–333. 32393826 10.1038/s41584-020-0413-5 PMC7987137

[32] Z. Wu , D. Ma , H. Yang , et al., “Fibroblast‐Like Synoviocytes in Rheumatoid Arthritis: Surface Markers and Phenotypes,” International Immunopharmacology 93 (2021): 107392–107399. 33529910 10.1016/j.intimp.2021.107392

[33] Y. Sun , N. Li , Y. Cai , X. Zhao , and H. Yang , “The Polymethoxylated Flavone Hexamethylquercetagetin Suppresses NF‐κB Signaling and Inhibits Cell Survival in Cervical Carcinoma,” Growth Factors 41, no. 1 (2023): 1–7. 36371694 10.1080/08977194.2022.2144282

[34] R. Tian , X. Li , Y. Li , K. Wang , C. Wang , and P. Yang , “1,25(OH)_2_D_3_ Promotes Chondrocyte Apoptosis and Restores Physical Function in Rheumatoid Arthritis Through the NF‐κB Signal Pathway,” Biomedicine & Pharmacotherapy 106 (2018): 149–155. 29957465 10.1016/j.biopha.2018.06.061

[35] E. Jimi , F. Huang , and C. Nakatomi , “NF‐κB Signaling Regulates Physiological and Pathological Chondrogenesis,” International Journal of Molecular Sciences 20, no. 24 (2019): 6275–6285. 31842396 10.3390/ijms20246275 PMC6941088

[36] S. Liu , H. Ma , H. Zhang , C. Deng , and P. Xin , “Recent Advances on Signaling Pathways and Their Inhibitors in Rheumatoid Arthritis,” Clinical Immunology 230 (2021): 108793–108821. 34242749 10.1016/j.clim.2021.108793

[37] Y. Jia , B. Feng , X. Ji , et al., “Complement Factor H Attenuates TNF‐α‐induced Inflammation by Upregulating EIF3C in Rheumatoid Arthritis,” Journal of Translational Medicine 21, no. 1 (2023): 846. 37996918 10.1186/s12967-023-04730-2 PMC10668393

[38] J. Chen , W. Wu , M. Zhang , and C. Chen , “Taraxasterol Suppresses Inflammation in IL‐1β‐induced Rheumatoid Arthritis Fibroblast‐Like Synoviocytes and Rheumatoid Arthritis Progression in Mice,” International Immunopharmacology 70 (2019): 274–283. 30851708 10.1016/j.intimp.2019.02.029

[39] X. Wang , Y. Kong , and Z. Li , “Advantages of Chinese Herbal Medicine in Treating Rheumatoid Arthritis: A Focus on Its Anti‐Inflammatory and Anti‐Oxidative Effects,” Frontiers in Medicine 11 (2024): 1371461. 38515982 10.3389/fmed.2024.1371461 PMC10954842

[40] H. Wang , Z. Wang , L. Wang , et al., “IL‐6 Promotes Collagen‐Induced Arthritis by Activating the NLRP3 Inflammasome Through the Cathepsin B/S100A9‐Mediated Pathway,” International Immunopharmacology 88 (2020): 106985–106994. 33182050 10.1016/j.intimp.2020.106985

[41] Y. J. Yang , L. J. Lu , J. J. Wang , et al., “Tubson‐2 Decoction Ameliorates Rheumatoid Arthritis Complicated With Osteoporosis in CIA Rats Involving Isochlorogenic Acid A Regulating IL‐17/MAPK Pathway,” Phytomedicine 116 (2023): 154875–154891. 37263000 10.1016/j.phymed.2023.154875

[42] W. Li , K. Wang , Y. Liu , et al., “A Novel Drug Combination of Mangiferin and Cinnamic Acid Alleviates Rheumatoid Arthritis by Inhibiting TLR4/NFκB/NLRP3 Activation‐Induced Pyroptosis,” Frontiers in immunology 13 (2022): 912933. 35799788 10.3389/fimmu.2022.912933 PMC9253268

[43] R. Zhang , L. Han , W. Lin , et al., “Mechanisms of NLRP3 Inflammasome in Rheumatoid Arthritis and Osteoarthritis and the Effects of Traditional Chinese Medicine,” Journal of Ethnopharmacology 321 (2024): 117432. 37992880 10.1016/j.jep.2023.117432

[44] Y. He , B. Cheng , B. J. Guo , et al., “Metabonomics and 16S rRNA Gene Sequencing to Study the Therapeutic Mechanism of Danggui Sini Decoction on Collagen‐Induced Rheumatoid Arthritis Rats With Cold Bi Syndrome,” Journal of Pharmaceutical and Biomedical Analysis 222 (2023): 115109. 36270097 10.1016/j.jpba.2022.115109

[45] L. Liu , T. Li , H. Dong , and X. Wang , “High‐Throughput Metabolomics Integrated Network Pharmacology Reveals the Underlying Mechanism of Paeoniae Radix Alba Treating Rheumatoid Arthritis,” Molecules 27, no. 20 (2022): 7014. 36296605 10.3390/molecules27207014 PMC9609690

[46] M. Yue , Y. Tao , Y. Fang , et al., “The Gut Microbiota Modulator Berberine Ameliorates Collagen‐Induced Arthritis in Rats by Facilitating the Generation of Butyrate and Adjusting the Intestinal Hypoxia and Nitrate Supply,” The FASEB Journal 33, no. 11 (2019): 12311–12323. 31425655 10.1096/fj.201900425RR PMC6902671

[47] J. Qiu , B. Wu , S. B. Goodman , G. J. Berry , J. J. Goronzy , and C. M. Weyand , “Metabolic Control of Autoimmunity and Tissue Inflammation in Rheumatoid Arthritis,” Frontiers in immunology 12 (2021): 652771. 33868292 10.3389/fimmu.2021.652771 PMC8050350

[48] G. van Hall , A. Steensberg , C. Fischer , et al., “Interleukin‐6 Markedly Decreases Skeletal Muscle Protein Turnover and Increases Nonmuscle Amino Acid Utilization in Healthy Individuals,” Journal of Clinical Endocrinology & Metabolism 93, no. 7 (2008): 2851–2858. 18430776 10.1210/jc.2007-2223

[49] A. Anbanandam , D. C. Albarado , D. C. Tirziu , M. Simons , and S. Veeraraghavan , “Molecular Basis for Proline‐ and Arginine‐Rich Peptide Inhibition of Proteasome,” Journal of Molecular Biology 384, no. 1 (2008): 219–227. 18823992 10.1016/j.jmb.2008.09.021 PMC2632303

