# Supplementary material for: Integration of Bioinformatics, Serum Pharmacochemistry, and Metabolomics to Reveal the Mechanisms of Danggui Buxue Decoction in Anti‐Rheumatoid Arthritis Through Inflammation and NF‐κB Signaling Pathway Regulation
Source: Immun Inflamm Dis. 2025 Sep 16;13(9):e70259. doi: 10.1002/iid3.70259 (PMC12439196; doi:10.1002/iid3.70259)
Supplement: Supplementary file 1 — Supplementary materials. [file IID3-13-e70259-s001.zip › Supplementary materials/Supplementary materials.docx]

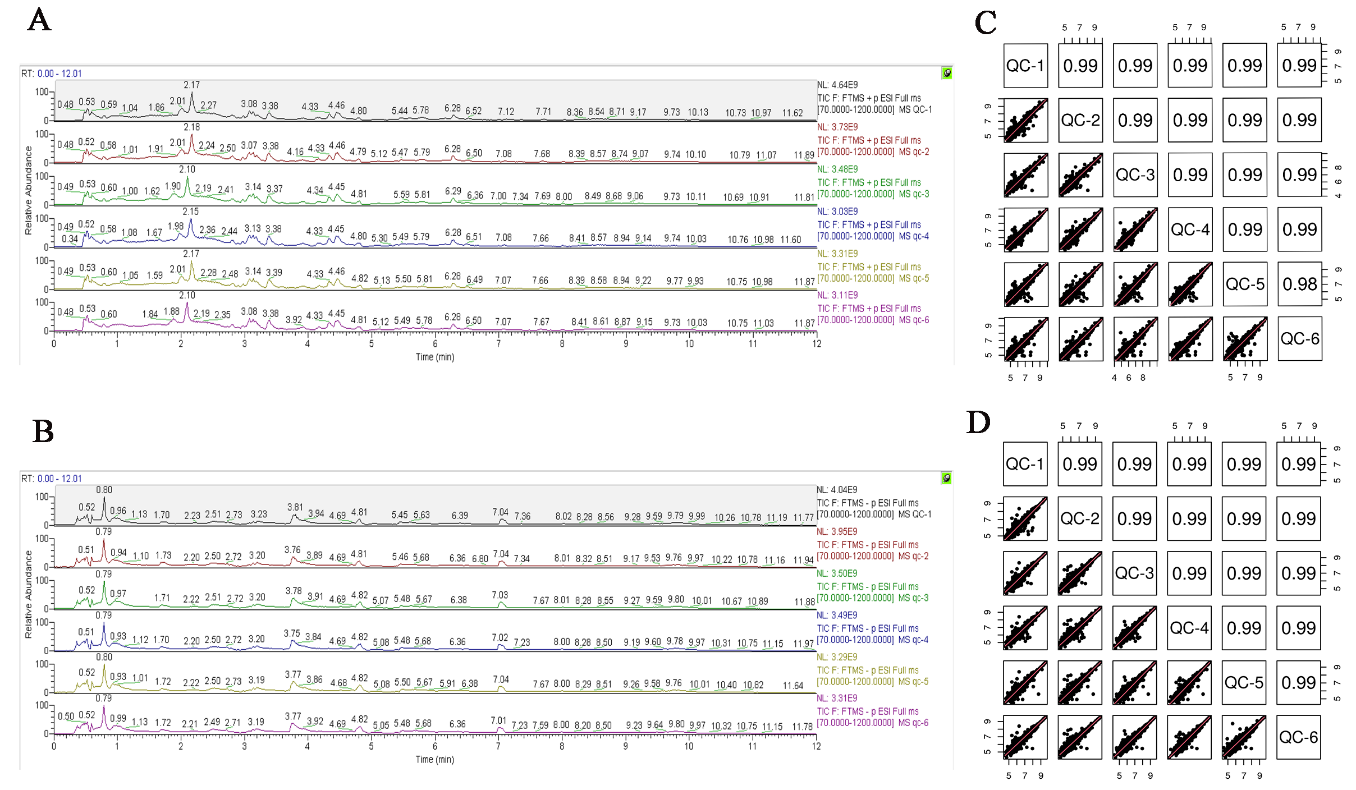


**Figure S1** The TIC of the QC samples in (A) positive and (B) negative ion modes. The pearson correlation coefficients between the QC samples in (C) positive and (D) negative modes.
